# Mycotoxin exposure through the consumption of processed cereal food for children (< 5 years old) from rural households of Oshana, a region of Namibia

**DOI:** 10.1007/s12550-024-00580-z

**Published:** 2025-01-14

**Authors:** Maria A. Angula, Anthony Ishola, Muvari Tjiurutue, Michael Sulyok, Rudolf Krska, Chibundu N. Ezekiel, Jane Misihairabgwi

**Affiliations:** 1 Department of Human, Biological, and Translational Medical Sciences, School of Medicine, University of Namibia, Windhoek, Namibia; 2 Department of Pharmaceutical Sciences, School of Pharmacy, Faculty of Health Sciences and Veterinary Medicine, University of Namibia, Windhoek, Namibia; 3 Department of Biochemistry, Microbiology and Biotechnology, School of Science, University of Namibia, Windhoek, Namibia; 4 Institute of Bioanalytics and Agro-Metabolomics, Department of Agrobiotechnology (IFA-Tulln), University of Natural Resources and Life Sciences, Konrad Lorenz Str. 20, 3430 Vienna, Tulln Austria; 5 Institute for Global Food Security, School of Biological Sciences, Queen’S University Belfast, University Road, Belfast, Northern Ireland BT7 1NN UK

**Keywords:** Mycotoxin exposure, Processed cereal food, 3-Nitropropionic acid, Aflatoxin, Margin of exposure, Risk assessment

## Abstract

Mycotoxin exposure from contaminated food is a significant global health issue, particularly among vulnerable children. Given limited data on mycotoxin exposure among Namibian children, this study investigated mycotoxin types and levels in foods, evaluated dietary mycotoxin exposure from processed cereal foods in children under age five from rural households in Oshana region, Namibia. Mycotoxins in cereal-based food samples (*n* = 162) (*mahangu* flour (*n* = 35), sorghum flour (*n* = 13), *mahangu* thin/thick porridge (*n* = 54), *oshikundu* (*n* = 56), and *omungome* (*n* = 4)) were determined by liquid chromatography-tandem mass spectrometry. Aflatoxin B_1_ (AFB_1_, 35.8%), zearalenone (27.2%), fumonisin B_1_ (FB_1_, 24.1%), citrinin (CIT, 12.4%) and deoxynivalenol (10.5%) were the major mycotoxins quantified. Food samples (35.8% (*n* = 58) and 6.2% (*n* = 10)) exceeded the 0.1 µg/kg AFB_1_ and 200 µg/kg FB_1_ EU limit for children’s food, respectively. Several emerging mycotoxins including the neurotoxic 3-nitropropionic acid, moniliformin (MON), and tenuazonic acid were quantified in over 50% of all samples. Co-occurrence of AFB_1_, CIT, and FB_1_ detected in 4.9% (*n* = 8) samples, which could heighten food safety concerns. Regarding exposure assessment and risk characterization, average probable dietary intake for AFB_1_ from all ready-to-eat-foods was 0.036 µg/kg bw/day, which resulted in margin of exposures (*MOE*) of 11 and 0.65 risk cancer cases/year/100,000 people, indicating a risk of chronic aflatoxicosis. High tolerable daily intake values for FB_1_, and *MOE* for beauvericin and MON exceeded reference values. Consumption of a diversified diet and interventions including timely planting and harvesting, best grain storage, and other standard postharvest food handling practices are needed to mitigate mycotoxin exposure through contaminated cereal foods and to safeguard the health of the rural children in Namibia.

**Supplementary Information:**

The online version contains supplementary material available at 10.1007/s12550-024-00580-z.

## Introduction

Namibia, a country in the southern part of sub-Saharan Africa, grapples with climatic challenges such as high temperatures and recurrent droughts (Awala et al. 2019). These conditions foster fungal proliferation in agricultural food commodities, posing significant health risks, particularly to vulnerable populations like children. Contamination of food commodities by filamentous fungi gives rise to mycotoxins, which are toxic secondary metabolites known for their adverse health effects in humans. Major mycotoxins of significant health concern include aflatoxin (AF, primarily produced by *Aspergillus* species), fumonisins, deoxynivalenol (DON), and zearalenone (ZEN, produced by various *Fusarium* species), as well as ochratoxins (produced by *Aspergillus* and *Penicillium* species) (Doughari et al. 2015; Misiharabgwi et al. 2023).

The occurrence of mycotoxins is widely reported in agricultural commodities and processed food products (Chilaka and Mally 2020). Thus, this underscores the significance of food ingestion as the main route of human exposure to mycotoxins in comparison with inhalation and dermal contact (CAST 2003; Alvito et al. 2022). Moreover, mycotoxins exhibit resistance to common food processing and cooking methods, remaining toxic even at low concentrations (Wasseem et al. 2015). Children are more vulnerable to mycotoxin exposure due to their frequent consumption of cereal-based foods relative to their body weight, higher metabolic rate, and immature detoxification systems (Lombard et al. 2014; Peraica et al. 2014; Alvito et al. 2022). Particularly in rural households of Namibia’s Oshana region, where staple cereal consumptions are predominant (Angula et al. 2024a), determining the risk of mycotoxin contamination and exposure among children’s population is imperative.

Chronic exposure to mycotoxins in low doses can lead to severe health implications, including liver and esophageal cancers, kidney diseases, neural tube defects, and immunotoxic effects, in both adults and children (Wu et al. 2024; Kowalska et al. 2016). Additionally, studies conducted globally have associated mycotoxins with adverse health effects in children; the effects include stunting, wasting, developmental defects, increased mortality, and morbidity (Abdulrazzaq et al. 2004; Shirima et al. 2015a, b; Gong et al. 2016; Chen et al. 2018; Kimanya et al. 2021).

Similar to the EU, some African countries have set regulatory limits for controlling mycotoxins in food although these countries still adopt the EU limits for trading purposes (Chilaka and Mally 2020). The EU has set maximum allowable limits for AFB_1_ at 0.10 µg/kg, and for fumonisin B_1_ (FB_1_), total fumonisins and deoxynivalenol (DON) at 200 µg/kg (EC 2006). On the other hand, the Codex Alimentarius — Codex STAN 193 sets international maximum limits for total aflatoxins (5 µg/kg for cereal-based foods intended for children), fumonisins B_1_ + B_2_ (2000 µg/kg) and deoxynivalenol (200 µg/kg) in food products (Codex Alimentarius Commission 1995). However, some countries including Namibia still lack specific national regulations for mycotoxin control, making reliance on international standards crucial for ensuring food safety. Previous studies conducted in Namibia have revealed contamination of cereal-based foods, including those derived from sorghum (*Sorghum bicolor*) and pearl millet (*Pennisetum glaucum* (L) R (BR)), commonly known as mahangu in Namibia (Misihairabgwi et al. 2018; Nafuka et al. 2019; Kaela et al. 2023). In a large portion of the examined foods, total aflatoxins and FB_1_ levels surpassed the maximum limits of 5 μg/kg and 2000 μg/kg set by Codex Alimentarius (1995) as well as the 0.10 µg/kg and 200 µg/kg EU limits for AFB_1_ and fumonisins, respectively (EC 2006). Similarly, other studies in Africa have reported alarming levels of mycotoxin contamination in various cereal foods (Ojuri et al. 2018; Ezekiel et al. 2021; Tshalibe et al. 2020; Ayeni et al. 2023), indicating a widespread health concern, particularly for children who consume the foods.

Despite the EU’s establishment of maximum limits for certain mycotoxins to protect the consumers, global studies continue to reveal children’s exposure to these toxins at levels exceeding recommended limits. In South Africa, Tshalibe et al. (2020) reported exposure of children from Eastern Cape province to high levels of fumonisins, DON and ZEN. Studies from Nigeria also reported the exposure of infants and young children to aflatoxins, fumonisins, beauvericin (BEAU), and/or moniliformin (MON) with exposure levels either exceeding the established limits or falling at a low margin of exposure; an indicator of health risk to children (Adetunji et al. 2017; Ojuri et al. 2018; Ezekiel et al. 2021; Ayeni et al. 2023). Furthermore, a study from Tanzania also reported the co-exposure of children to aflatoxins, DON, and FB_1_, with the exposure levels of aflatoxins ranging 1–786 ng/kg bw/day and exceeding the 0.017 ng/kg bw/day threshold by many folds (Kimanya et al. 2014). Another study from Tanzania indicated that infants and young children were exposed to aflatoxins, DON and fumonisins through the consumption of contaminated maize-based complementary foods at levels exceeding the EU established limits (Kamala et al. 2017). Mycotoxin exposure in children through consumption of breakfast cereals was also reported in other parts of the world (Iqbal et al. 2014; Assuncao et al. 2015; Foerster et al. 2022). This suggests the necessity for mitigation strategies to protect this group of vulnerable populations.

Limited data exists regarding mycotoxin exposure in children in Namibia, highlighting a crucial knowledge gap. Addressing this gap is imperative to inform policymakers of the dangers of mycotoxin exposure, establish appropriate standards, and devise mitigation strategies. Therefore, this study aimed to assess the types and levels of mycotoxins present in processed cereal flours and their ready-to-eat foods consumed by children under 5 years of age and evaluate mycotoxin exposure among the children from rural households in Oshana region, Namibia.

## Materials and methods

### Study setting

This study was conducted in rural households of the Oshana region in Namibia. Located in central northern Namibia, the region experiences hot daytime temperatures averaging above 33 °C and cold winter nights with temperatures as low as 8 °C. Despite its erratic rainfall, rural households in Oshana region rely heavily on subsistence farming for their food supply (Awala et al. 2019; Oshana Regional Council 2023).

### Research design and food sample collection

This study was conducted in Oshana rural households employing a cross-sectional quantitative design between April and November 2023. A total of 162 food samples including processed cereal flours (*mahangu* flour (*n* = 35) and sorghum flour (*n* = 13)) and their ready-to-eat foods (*mahangu* thin/thick porridge (*n* = 54), traditional beverage (*oshikundu*) (*n* = 56), and Mahangu cake (*omungome*) (*n* = 4)) were collected from rural households. The food samples were collected from the portions intended for preparation (in the case of flour) and direct consumption as ready-to-eat foods by children under age 5 who are not actively breastfeeding. Flour samples weighed 200–300 g and collected from 1 to 2 kg stored flour portions, while ready-to-eat food samples (25–35 g) were collected from 250 to 500 g freshly prepared plate portions. Processed cereal flours were collected in brown paper bags, whereas 50 mL Falcon tubes were used to collect the ready-to-eat foods. Generally, one food sample was collected per household with one child under age 5. In households with more than one under 5-year-old children, one sample per food type was collected for children who consumed the same food. This was necessary because food preparation in the participating rural households is typically communal with all children sharing the same meal prepared in a single pot, thereby reflecting the consistent dietary mycotoxin exposure for all children within each household. All samples were refrigerated at 4 °C at the sampling site and then transferred to − 20 °C within 24 h until mycotoxin analysis.

### Population size calculation

Population size was calculated based on previously established formula (Naing et al. 2006) as: *n* = [Z2 *P* (1 − *P*)] / d2; whereby *n* = population size, *Z* = *Z* statistic for a level of confidence (1.96 at confidence level of 95%), *P* = 0.123, based on Oshana population Census (Namibia Population and Housing Census 2011), which reported that 12.3% of the population in Oshana region are children (0–4 years) and *d* = precision (0.05). Given that 5% of children are 0–11 months old, *P* = 0.073. Therefore, *n* = 1.96 × 1.96 × 0.073(1 − 0.073) / (0.05 × 0.05) = 104 children. However, a total of 248 children were recruited to participate in this study.

### Multi-mycotoxin analysis of food samples

Multiple mycotoxins in processed cereal flours and their ready-to-eat food samples were determined using the dilute and shoot LC–MS/MS method, as described by Sulyok et al. (2020). The method encompasses the analysis of more than 500 secondary metabolites originating from bacteria, fungi, plants, and of unspecified origins.

### Reagents and sample preparation

All chemicals, reagents, and sample preparation procedures were used as previously outlined by Angula et al. (2024b). Briefly, 5 g of a food sample was weighed into a 50-mL Falcon tube (Sarstedt, Nümbrecht, Germany) and homogenized with 20 mL of acetonitrile/water/acetic acid 79:20:1 (v/v/v). Mycotoxins were then extracted from the samples by shaking on the GFL 3017 rotary shaker (GFL, Burgwedel, Germany) for 90 min. Thereafter, the extracts were diluted in acetonitrile/water/acetic acid 20:79:1 (v/v/v) in a 1:1 (v/v) ratio, and 5 μL of the diluted extract was injected into the LC–MS/MS instrument.

### LC–MS/MS parameters

The screening for mycotoxins and other metabolites was conducted using a QTrap 5500 LC–MS/MS system (Applied Biosystem, Foster City, CA, USA) equipped with a TurboIonSpray electrospray ionization (ESI) source, paired with a 1290 Series HPLC system (Agilent, Waldbronn, Germany), as previously described by Sulyok et al. (2020). Chromatographic separation took place at 25 °C using a Gemini® C18 column (150 × 4.6 mm i.d., 5-μm particle size) with a C18 security guard cartridge (4 × 3 mm i.d.) from Phenomenex (Torrance, CA, USA). The details of the chromatographic method, as well as the mass spectrometric parameters, followed the procedures described by Sulyok et al. (2020). The apparent recoveries of analytes were consistent with those established during the validation for the ingredient base of ready-to-use therapeutic foods (Malachova et al. 2014; Sulyok et al. 2020, 2024). Similarly, the overall method performance data were utilized, as described in previous studies (Misihairabgwi et al. 2018; Nafuka et al. 2019). ESI–MS/MS analysis was carried out in time-scheduled multiple reaction monitoring (MRM) mode, scanning two fragmentation reactions per analyte, in both positive and negative polarities, across two separate chromatographic runs per sample. The MRM detection window was set around each analyte’s expected retention time, with a tolerance of ± 20 s for the positive mode and ± 26 s for the negative mode. Positive analytes were confirmed by two MRM transitions per analyte, yielding 4.0 identification points as per European Commission (EC) decision 2002/657 (EC 2002). Additionally, the retention times and intensity ratios for the two MRM transitions matched those of an authentic standard within 0.03 min and 30%, respectively.

### Quantification of metabolites

Metabolite quantification was performed using linear calibration curves, weighted by 1/x, with serial dilutions from an external multi-component stock solution. The weighted calibration curve approach was adopted to address heteroskedasticity observed in the data, as residual variability increased with analyte concentration. Concentrations were adjusted for apparent recoveries. The limits of detection (*LOD*) and quantification (*LOQ*) were determined based on the standard deviation of samples spiked at low concentrations, following the guidelines outlined in the EURACHEM guide (Magnusson and Ornemark 2014). A previously established general expanded measurement uncertainty of 50% (coverage factor *k* = 2) was applied to the method (Stadler et al. 2018). To ensure quality control, the study regularly participated in a proficiency testing program organized by BIPEA (Gennevilliers, France), which covered a range of food matrices including grains, nuts, dried fruits, and baby food, with 96% of *z*-scores falling between − 2 and + 2 (Sulyok et al. 2020). Additionally, a commercially available quality control material from Romer Labs (Tulln, Austria) consisting of a naturally contaminated wheat sample with DON, T-2 toxin, HT-2 toxin, and ochratoxin A was extracted and included in each analytical sequence.

### Dietary mycotoxin exposure and risk assessment estimations

#### Chronic dietary exposure assessment by deterministic approach

Mycotoxin exposure assessment of children under age five who consume the sampled foods was conducted using the deterministic approach and a point estimate of the mycotoxins, following guidelines established by EFSA (2007). This involved estimating the average probable daily intake (*APDI*) of dietary mycotoxins in the children by utilizing data on mean mycotoxin levels in the ready-to-eat foods alongside the average amount of food ingested by the children per unit of average body weight. Only mycotoxin data for the ready-to-eat foods were utilized in the exposure estimates since the flour samples will require dilution and cooking before consumption and these may impact the mycotoxin concentrations (Ezekiel et al. 2019). The *APDI* calculations were performed using the following formula:

$$APDI=\left[mean \;mycotoxin \;concentration\left(\mu g/kg\right)\times \;average \;food \;consumption \left(kg/day\right)\right]/mean \;body \;weight \left(kg\right)$$

In order to estimate the average daily food consumption (kg/day) per child, the weight of food consumed by each child during a single meal was measured using a portable electronic weighing scale and multiplied by the number of feeding times per day. This method provides a baseline estimate but introduces uncertainties, as it assumes uniformity in food types and quantities across meals. Children in this study mostly consume staple cereals including mahangu thick/thin porridge and *oshikundu*. The food consumption pattern data of the children is available in a separate study by Angula et al. (2024a). Quality control measures included training for data collectors and usage of calibrated weighing scale for food measurements to ensure accuracy in the data collected. Similarly, the body weight (kg) of each child was taken using a calibrated portable electronic digital scale. The weight was taken to the nearest 0.1 kg, with 2–3 measurements obtained to ensure accuracy. The mean weight (kg) of the 248 children was then calculated and applied in the *APDI* estimation. Other details relating to the recruitment, consent, and ethical approval for involvement of the 248 children were already published (Angula et al. 2024a). It is important to highlight that the *APDI* is the recommended estimate for long-term population exposure assessments by EFSA (2007) and IPCS (2020).

Handling data below the limit of detection (*LOD*), often referred to as left-censored data, is critical for exposure assessment and subsequent risk assessment (EFSA 2010; IPCS 2020). In cases where the proportion of left-censored data exceeded 60% in the datasets, lower bound (LB) and upper bound (UB) scenarios were applied, as recommended by IPCS (2020) and EFSA (2010). The LB scenario involved substituting mycotoxin concentrations < *LOD* with a value of 0 (the minimum possible value), while the UB scenario substituted mycotoxin concentrations < *LOD* with the *LOD* value (the maximum possible value). In this study, an UB/LB case-scenario was assumed to estimate the exposure of children to BEAU, citrinin (CIT), DON, FB_1_, and ZEN (IPCS/GEMS 1995; EFSA 2010). In instances where less than 60% of the datasets contained levels below the *LOD*, the exposure was estimated by substituting *LOD*/2 (the middle bound) for these values (EFSA 2010). In the present study, *LOD*/2 was assigned for aflatoxins and MON. The *omungome* (*n* = 4) was excluded from the exposure assessment and risk characterization due to their sample size and low contamination levels in this food type.

#### Risk characterization of mycotoxins

Risk characterization for aflatoxin, a genotoxic and carcinogenic compound, was based on the margin of exposure (MOE) (EFSA 2007). The *MOE* was calculated by dividing the benchmark dose lower confidence limit (BMDL_10_) of 0.4 µg/kg bw/day established for AFB_1_ from rodent data (EFSA 2020b) with the estimated APDI. The risk level of aflatoxins was determined by assessing the magnitude of the *MOE*. *MOE* of 10,000 or more indicated no risk and was considered of low public health concern (European Food Safety Authority 2014), whereas *MOE* values below and farther away from 10,000 indicated a public health risk. The conservative approach of using the BMDL_10_ of 0.4 µg/kg bw/day for AFB_1_ to estimate the MOE for total aflatoxins was adopted in line with EFSA (2020) recommendations of assuming equal potencies for all compounds.

The risk of mycotoxin exposure to non-genotoxic and non-carcinogenic mycotoxins such as BEAU, CIT, DON, fumonisins, MON, and ZEN were characterized by comparing the calculated *APDI*s for each mycotoxin to their respective established reference points or health-based guidance values (HBGVs)/tolerable daily intake (*TDI*) values (EFSA 2012, 2014, 2018; JECFA 2017). For total fumonisins, a *TDI* of 1 µg/kg bw/day was adopted (EFSA 2018), whereas *TDI*s of 1 µg/kg bw/day and 0.25 µg/kg bw/day were applied for DON and ZEN, respectively (EFSA 2012). The risk from CIT exposure was assessed using a level of no concern for nephrotoxicity (0.2 μg/kg bw/day) due to the absence of an established HBGV resulting from substantial uncertainties in available toxicity data (EFSA 2012). For BEAU and MON, the lowest dose of 90 μg/kg bw/day and BMDL_05_ of 200 μg/kg bw/day were applied as reference points for calculating their *MOE*s (EFSA 2014, 2018).

#### Estimation of risk to primary liver cancer due to consumption of food

Aflatoxin B_1_ (AFB_1_) is associated with the development of liver cancer in humans, and it has been reported to synergistically interact with the hepatitis B virus (HBV), thus, increasing the chances of hepatocellular carcinoma (HCC) by 30% (FAO/WHO 1998). The risk of developing liver cancer among rural children in the Oshana region due to AFB_1_ exposure through contaminated food was estimated per 100,000 population per year. This estimation was obtained by multiplying the *APDI* values with the average HCC potency figure derived from individual potencies of HBsAg-positive (0.3) and HBsAg-negative groups (0.01) (FAO/WHO 1998; EFSA 2007), and the HBV infection rates among children in Namibia (0.027) (Mhata et al. 2017). The formula used for calculating the children population risk is as follows:

$$Children \;population \;risk=APDI\times Average \;potency$$

whereby the average potency is determined by:

$$Average \;potency=\left(\left(0.3\times 0.027\right)+\left(0.01\times 0.973\right)\right)=0.0178 \;cancers \;per \;year \;per\text{ 100,000 }per ng/\left(kg bw\right)/day {AFB}_{1}$$

### Research ethics

This research was approved by the Decentralized Ethics Committee of the University of Namibia (Reference number: DEC04/2022), the Ministry of Health and Social Services (Reference number: 22/4/2/3) and National Commission on Research, Science and Technology (Authorization number: 202306006). The research followed the Declaration of Helsinki. Oshana regional councilors and the village heads granted permission to enter the rural communities. The research objectives were explained to the regional councilors, village heads, and the whole community. Ethical issues pertaining to confidentiality, voluntary participation, withdrawal from the study, and risk/benefits of the study were explained to the children care providers. Each childcare provider signed the informed consent prior to participation in the study.

### Data analysis

All data were analyzed using IBM® SPSS® Statistics version 27. Initially, the data was assessed for normality using the Kolmogorov–Smirnov test. Since the data did not follow a normal distribution, the Kruskal–Wallis test, a non-parametric method was used to determine if there was a significant difference in the distribution of mycotoxin levels among various types of processed cereal foods. Descriptive statistics was then used to represent the data.

## Results and discussion

### Demographic characteristics of under 5-year-old children from Oshana region

Table 1 presents body weight and food consumption data for the 248 children under the age of five in Oshana rural households. The average body weight of these children was 12.5 kg, while the average amount of food consumed was 0.43 kg. These figures align with the data published for children in Nigeria (Adetunji et al. 2017; Ezekiel et al. 2021).

**Table 1:** Body weight and food consumption data of under 5-year-old children from Oshana region (*n* = 248)

Variable	*Mean*	Standard deviation	Range	*Median*
Body weight (kg)	12.49	2.34	6.2–18.65	12.35
Amount of food consumed per day (kg)	0.43	0.082	0.25–0.5	0.5

*n* number of children recruited in the study, *kg* kilogram

### Occurrence and level of multi-metabolites in food samples

A total of 188 metabolites, comprising 168 fungal, two bacterial, three plant, and 15 unspecified metabolites were quantified in 162 food samples (Table S1). The food samples encompassed processed cereal flours such as *mahangu* flour (21.6%) and sorghum flour (8.0%), as well as ready-to-eat cereal foods including *mahangu* thin/thick porridge (33.3%), *oshikundu* (34.6%), and *omungome* (2.5%). Among the 168 fungal metabolites quantified, 22 were identified as major mycotoxins and their derivatives (Tables 2 and 3), while others comprised unregulated metabolites such as those from *Fusarium*, *Aspergillus*, *Penicillium*, and *Alternaria* (Table 5).

**Table 2:** Major mycotoxins and their derivatives quantified in flour samples from Oshana rural households

Mycotoxins	All flour samples *n* = 48				*Mahangu n* = 35				Sorghum *n* = 13			
	*Np*	%*p*	Range	*Mean* ± *SD*	*Np*	%*p*	Range	*Mean* ± *SD*	*Np*	%*p*	Range	*Mean* ± *SD*
Aflatoxin B_1_	16	33.3	0.19–22.0	4.11 ± 5.78	5	14.3	0.19–2.23	0.93 ± 0.86	11	84.6	0.29–22.0	5.56 ± 6.52
Aflatoxin B_2_	4	8.3	0.26–0.75	0.45 ± 0.22	< LOD	< LOD	< LOD	< *LOD*	4	30.8	0.26–0.75	0.45 ± 0.22
Aflatoxin G_1_	3	6.3	0.19–0.44	0.31 ± 0.12	3	8.6	0.19–0.44	0.31 ± 0.12	< *LOD*	< *LOD*	< *LOD*	< *LOD*
Total aflatoxins	16	33.3	0.2–22.5	1.43 ± 3.89	5	14.3	0.19–2.23	0.16 ± 0.49	11	84.6	0.29–22.5	4.84 ± 6.43
Aflatoxin M_1_	1	2.1	0.59	0.59	< *LOD*	< *LOD*	< *LOD*	< *LOD*	1	7.7	0.59	0.59
Fumonisin A_1_	5	10.4	2.18–6.41	3.90 ± 1.54	5	14.3	2.18–6.41	3.90 ± 1.54	< *LOD*	< *LOD*	< *LOD*	< *LOD*
Fumonisin A_2_	1	2.1	1.57	1.57	1	2.9	1.57	1.57	< *LOD*	< *LOD*	< *LOD*	< *LOD*
Fumonisin B_1_	8	16.7	14.5–281	110 ± 96.7	8	22.9	14.5–281	110 ± 96.9	< *LOD*	< *LOD*	< *LOD*	< *LOD*
Fumonisin B_2_	8	16.7	9.80–101	38.6 ± 28.5	7	20	9.80–101	40.5 ± 30.3	1	7.7	25.53	25.5
Fumonisin B_3_	5	10.4	12.9–46.5	24.3 ± 15.3	5	14.3	12.9–46.5	24.3 ± 15.3	< *LOD*	< *LOD*	< *LOD*	< *LOD*
Fumonisin B_4_	5	10.4	10.2–29.1	15.14 ± 8.10	4	11.4	10.2–29.1	16.4 ± 8.78	1	7.7	10.2	10.2
Total fumonisins	9	18.8	0.00–451	29.3 ± 88	8	22.9	14.5–451	39.2 ± 101	1	7.7	35.7	35.7
Zearalenone	9	18.8	0.30–6.14	1.36 ± 1.86	4	11.4	0.58–1.88	0.99 ± 0.60	5	38.5	0.30–6.14	1.65 ± 2.53
Deoxynivalenol	2	4.2	26.9–114	70.3 ± 61.3	2	5.7	26.9–114	70.3 ± 61.3	< *LOD*	< *LOD*	< *LOD*	< *LOD*
Nivalenol	1	2.1	23.1	23.1	< *LOD*	< *LOD*	< *LOD*	< *LOD*	1	7.7	23.1	23.1
Monoacetoxyscirpenol	10	20.8	1.61–198	42.1 ± 61.3	1	2.9	60.3	60.3	9	69.2	1.61–198	40.1 ± 64.7
Diacetoxyscirpenol	7	14.6	1.93–50.6	14.3 ± 16.9	1	2.9	16.4	16.4	6	46.2	1.93–50.6	13.9 ± 18.5
Neosolaniol	5	10.4	1.94–11.6	5.45 ± 4.49	< *LOD*	< *LOD*	< *LOD*	< *LOD*	5	38.5	1.94–11.6	5.45 ± 4.49
Deacetylneosolaniol	4	8.3	71.3–215	115 ± 68.0	< *LOD*	< *LOD*	< *LOD*	< *LOD*	4	30.8	71.3–215	115 ± 68.0
T2-Tetraol	1	2.1	10.5	10.5	< *LOD*	< *LOD*	< *LOD*	< *LOD*	1	7.7	10.5	10.5
8-Acetylneosolanol	11	22.9	0.29–60.8	14.8 ± 19.8	1	2.9	6.38	6.38	10	76.9	0.29–60.8	15.6 ± 20.7
Citrinin	6	12.5	26.2–9703	1707 ± 3919	1	2.9	9703	9703	5	38.5	26.2–317	108 ± 120

Concentration in µg/kg

*n* number of samples analyzed, *np* number of positive samples, *%p* percent positive samples, *mean* positive samples only, *SD* standard deviation, < *LOD* less than limit of detection

**Table 3:** Major mycotoxins and their derivatives quantified in ready to eat food samples from Oshana rural households

All-ready to eat foods n = 114					*Oshikundu n* = 56				*Mahangu* thick/thin porridge *n* = 54				*Omungome n* = 4			
Metabolic type	*Np*	%*p*	Range	*Mean* ± *SD*	*Np*	%*p*	Range	*Mean* ± *SD*	*Np*	%*p*	Range	*Mean* ± *SD*	*Np*	%*p*	Range	*Mean* ± *SD*
Aflatoxin B_1_	42	36.8	0.16–40.1	2.67 ± 6.4	32	57.1	0.16–40.10	2.64 ±	10	18.5	0.2–13.2	2.75 ± 4.1	0	0.0	< *LOD*	< *LOD*
Aflatoxin B_2_	6	5.3	0.24–1.0	0.52 ± 0.3	3	5.4	0.30–1.02	0.61 ± 6.99	3	5.6	0.24–0.7	0.44 ± 0.2	0	0.0	< *LOD*	< *LOD*
Aflatoxin G_1_	1	0.9	1.53	1.53 ± 0	< *LOD*	< *LOD*	< *LOD*	< *LOD*	1	1.9	1.53	1.53 ± 0	0	0.0	< *LOD*	< *LOD*
Total aflatoxins	42	36.8	0.2–41.1	1.02 ± 4.2	32	57.1	0.16–41.1	1.54 ± 0.37	10	18.5	0.2–15.38	0.56 ± 2.30	0	0.0	< *LOD*	< *LOD*
Aflatoxin M_1_	3	2.6	0.64–1.8	1.20 ± 0.56	2	3.6	0.64–1.75	1.19 ± 0	1	1.9	1.22	1.22 ± 0	0	0.0	< *LOD*	< *LOD*
Fumonisin A_1_	9	7.9	0.73–46.8	12.4 ± 15.6	3	5.4	2.81–46.8	19.37 ± 0.7	6	11.1	0.73–29.81	8.87 ± 10.80	0	0.0	< *LOD*	< *LOD*
Fumonisin A_2_	2	1.8	13.5–35.6	24.53 ± 15.7	1	1.8	35.6	35.6 ± 23.9	1	1.9	13.5	13.46 ± 0	0	0.0	< *LOD*	< *LOD*
Fumonisin B_1_	31	27.2	8.43–1022	152.3 ± 251	14	25	8.43–1022	125.1 ± 0	17	31.5	9.80–609	174.8 ± 230	0	0.0	< *LOD*	< *LOD*
Fumonisin B_2_	17	14.9	8.22–523.93	100.7 ± 138.62	6	10.7	8.22–523.93	120.33 ± 281	11	20.4	8.59–308.14	89.95 ± 98.6	0	0.0	< *LOD*	< *LOD*
Fumonisin B_3_	9	7.9	13.44–163	71.23 ± 45.8	3	5.4	13.44–163	77 ± 203.3	6	11.1	31.82–112.7	68.34 ± 30.45	0	0.0	< *LOD*	< *LOD*
Fumonisin B_4_	8	7	18.12–157.64	58.37 ± 48.3	2	3.6	36.78–157.64	97.21 ± 77.3	6	11.1	18.12–101	45.42 ± 31.6	0	0.0	< *LOD*	< *LOD*
Total fumonisin	31	27.2	8.43–1949	67.57 ± 256.54	14	25	8.43–1949	53.44 ± 85.5	17	31.5	9.80–1111.4	87.22 ± 248.7	0	0.0	< *LOD*	< *LOD*
Zearalenone	35	30.7	0.25–40.16	5.46 ± 8.85	19	33.9	0.31–40.16	8.46 ± 273.3	15	27.8	0.25–16.71	1.99 ± 4.20	1	25	0.39–0.39	0.39
Deoxynivalenol	15	13.2	14.0–202.7	84.30 ± 62.4	5	8.9	39.7–107.13	80 ± 10.64	10	18.5	14.0–202.7	86.46 ± 75.8	0	0.0	< *LOD*	< *LOD*
Nivalenol	4	3.5	8.86–26.76	19.17 ± 7.47	3	5.4	8.86–26.76	18.76 ± 25.61	1	1.9	20.40	20.40 ± 0	0	0.0	< *LOD*	< *LOD*
Monoacetoxyscirpenol	31	27.2	1.41–111	19.44 ± 27.71	27	48.2	1.41–111	20.55 ± 9.1	4	7.4	5.3–16.74	12 ± 5.7	0	0.0	< *LOD*	< *LOD*
Diacetoxyscirpenol	6	5.3	1.46–3.92	2.12 ± 0.91	6	10.7	1.46–3.92	2.12 ± 29.54	0	0	< *LOD*	< *LOD*	0	0.0	< *LOD*	< *LOD*
Neosolaniol	1	0.9	4.44	4.44 ± 0	1	1.8	4.44–4.44	4.44 ± 0.91	0	0	< *LOD*	< *LOD*	0	0.0	< *LOD*	< *LOD*
Deacetylneosolaniol	2	1.8	16.5–44.1	30.29 ± 19.54	1	1.8	44.11–44.1	44.11 ± 0	1	1.9	16.5	16.5 ± 0	0	0.0	< *LOD*	< *LOD*
T2-Tetraol	5	4.4	11.12–74.71	32.67 ± 24.91	4	7.1	11.12–74.71	35.62 ± 0	1	1.9	21	21 ± 0	0	0.0	< *LOD*	< *LOD*
8-Acetylneosolanol	7	6.1	0.70–33.38	6.25 ± 12	6	10.7	1.25–33.38	7.17 ± 27.74	1	1.9	0.7	0.70 ± 0	0	0.0	< *LOD*	< *LOD*
Citrinin	14	12.3	23.85–1227	286.5 ± 344	8	14.3	61.76–1227	350.5 ± 13	6	11.1	23.85–733	201.1 ± 263	0	0.0	< *LOD*	< LOD

Metabolites concentration in µg/kg

*n* number of samples analyzed, *np* number of positive samples, %*p* percent positive samples, *mean* positive samples only, *SD* standard deviation, < *LOD* less than limit of detection

### Major mycotoxins and their derivatives in processed cereal food samples

This study revealed major mycotoxins and their derivatives present in cereal flour (Table 2) and ready-to-eat cereal foods (Table 3) consumed by under 5-year-old children from Oshana rural households. In cereal flour, the prevalent major mycotoxins included AFB_1_ (prevalence 33.3%; range 0.19–22.0 µg/kg), FB_1_ (prevalence 16.7%; range 14.5–281 µg/kg), ZEN (prevalence 30.7%; range 0.25–40.16 µg/kg), DON (prevalence 4.2%; range 26.9–114 µg/kg), and CIT (12.5%; range 26.2–9703 µg/kg). Similarly, in ready-to-eat foods, AFB_1_ (prevalence 36.8%; range 0.16–40.1 µg/kg), FB_1_ (prevalence 27.2%; range 8.43–1022 µg/kg), ZEN (prevalence 18.8%; range 0.30–6.14 µg/kg), DON (prevalence 13.2%; range: 14.0–202.7 µg/kg), and CIT (12.3%, range 23.85–1227 µg/kg) were most prevalent. Across all processed cereal food samples, major mycotoxins and their derivatives quantified (Table S2) included AFB_1_ prevalence 35.8%, range 0.16–40.1 µg/kg), total aflatoxin (prevalence 35.8%, range 0.16–41.1 µg/kg), FB_1_ (prevalence 24.1%, range 8.43–1022 µg/kg), total fumonisin (prevalence 24.7%, range 8.43–1949 µg/kg), ZEN (prevalence 27.2%, range 0.25–40.2 µg/kg), DON (prevalence 10.5%, range 14.0–203 µg/kg), monoacetoxyscirpenol (prevalence 25.3%, range 1.41–198 µg/kg), deacetylneosolaniol (prevalence 3.7%, range 16.5–215 µg/kg), and CIT (prevalence 12.4%, range 23.9–9703 µg/kg). Similar spectra of mycotoxins were identified in cereal food samples from previous studies conducted in Namibia, with the exception of the trichothecenes including deacetylneosolaniol and DON which were not previously reported (Angula et al. 2024a, b; Kaela et al. 2023; Misihairabgwi et al. 2018; Nafuka et al. 2019). Similar to our present study, DON was quantified in cereal flour samples in Nigeria, albeit at lower levels than observed herein (Ayeni et al. 2023; Ezekiel et al. 2019). *Omungome* was only contaminated with one major mycotoxin (ZEN).

Aflatoxins, which are known carcinogens, were present in all food samples except in a ready-to-eat cereal food (*omungome)*. The prevalence of AFB_1_ in the cereal flour samples was slightly higher than that of the ready-to-eat cereal foods. This disparity could be attributed to the smaller number of cereal flour samples analyzed in comparison with ready-to-eat foods. However, the mean values for AFB_1_ and total aflatoxins in flour samples were 1.5 times and 1.3 times higher than for ready-to-eat foods, respectively. Sorghum flour samples exhibited a high prevalence (84%) and a high *mean* ± *SD* level (5.56 ± 6.50 µg/kg) of AFB_1_ in comparison with *mahangu* flour (14.3%; 0.93 ± 0.86 µg/kg) and the ready-to-eat foods: *mahangu* thick/thin porridge (18.5%; 2.75 ± 0.23 µg/kg), and *oshikundu* (57.1%; 2.64 ± 6.99 µg/kg). The maximum AFB_1_ concentration of 40 µg/kg quantified in *oshikundu*, a ready-to-eat traditional cereal beverage was notably twice higher than that of sorghum flour and about 20 times higher than *mahangu* flour and *mahangu* thick/thin porridge, raising its safety concern. The Kruskal–Wallis test also indicated a significant difference in the distribution of AFB_1_ among the processed cereals (*P* < 0.05). The aflatoxin levels quantified in processed cereal foods in the present study are consistent with those reported in previous studies from Namibia (Angula et al. 2024b; Nafuka et al. 2019; Misihairabgwi et al. 2018), except for *oshikundu* samples, in which aflatoxins were not quantified in earlier research (Misihairabgwi et al. 2018). This reflects variability in raw materials and processing procedures for *oshikundu*. The quantification of aflatoxin in food has also been reported in other studies in other African countries; however, at levels higher than those observed in the present study (Ojuri et al. 2019; Wielogorska et al. 2019; Ayeni et al. 2023). Mycotoxin levels in flour were generally higher than levels in ready-to-eat foods; this finding agrees with previous work from Ezekiel et al. (2019), who reported as much as 129–383% higher mycotoxin levels in uncooked flour and plate-ready foods due to simple dilution. Mycotoxins are currently not regulated in Namibia. Consequently, this study applied EU limits as a reference for comparison. The study revealed that 33% of cereal flour and 37% of ready-to-eat food samples exceeded the 0.1 µg/kg EU limit for AFB_1_ in baby food (Table 4) (EC 2006). A similar trend was observed in Nigeria by Ayeni et al. (2023) for *ogi* and *tombran* complementary foods for infants and young children. The presence of aflatoxin in ready-to-eat foods exceeding maximum limits highlights food safety violation requiring immediate intervention.

**Table 4:** Number of food samples from Oshana rural households exceeding the EU threshold limits in children’s foods

Mycotoxin	EU limit (µg/kg)	All samples (*n* = 162) (%)	All flour samples (*n* = 48) (%)	All-ready to eat food samples *n* = 114 (%)
Aflatoxin B_1_	0.1	58 (35.8)	16 (33.3)	42 (36.8)
Fumonisin B_1_	200	10 (6.2)	2 (4.2)	8 (16.7)
Zearalenone	20	3 (1.9)	0	3 (2.6)
Deoxynivalenol	200	1 (0.6)	0	1 (0.9)

*n* total number of food samples analyzed (percentage)

Fumonisins were quantified in ready-to-eat foods, exclusively from *mahangu* thick/thin porridge and *oshikundu* at high mean and prevalence levels than the flour samples. *Mahangu* thick/thin porridge revealed high mean levels of FB_1_ (175 ± 230 µg/kg), which was 1.4 and 1.6 times higher than the levels in *oshikundu* (125 ± 281 µg/kg) and *mahangu* flour (110 ± 96.9 µg/kg), respectively. Statistically, there was a significant difference in the distribution of FB_1_, among the *mahangu* flour and *oshikundu* only (*P* < 0.05). The mean levels of fumonisins across all processed cereal food samples in this study were at least 1.2 times lower than the contamination levels reported by Angula et al. (2024b) and Nafuka et al. (2019) and 17.5 times lower than those documented by Misihairabgwi et al. (2018). At least 16.7% of the ready-to-eat foods and 4.2% of the cereal flour samples exceeded the 200 µg/kg EU fumonisin threshold limit in food intended for infants and young children (Table 4) (EC 2006). These mean fumonisin levels fall below those reported in complementary foods in Nigeria, Cameroon, and Tanzania (Kamala et al. 2017; Chuisseu et al. 2018; Ezekiel et al. 2021).

**Fig. 1:**
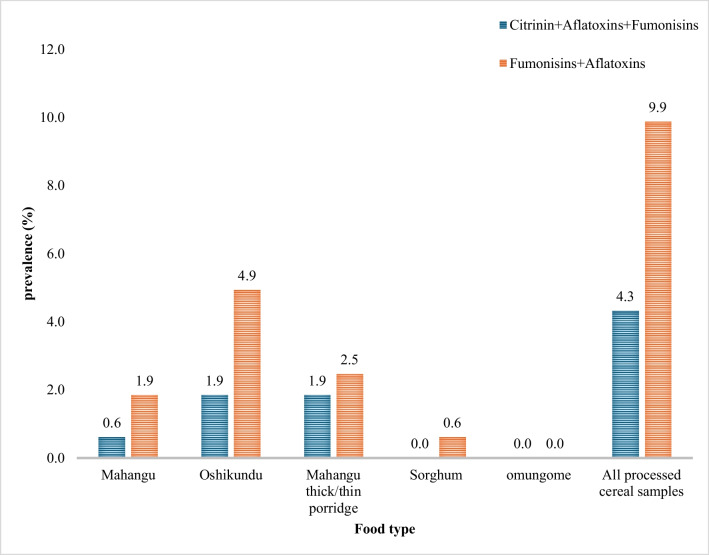
Co-occurrence of mycotoxins in cereal food samples

Citrinin was frequently quantified in cereal flour samples compared to ready-to-eat foods. *Mahangu* flour recorded the highest mean concentrations (9700 µg/kg), a thousand-fold greater than sorghum flour and the ready-to-eat foods (*mahangu* thick/thin porridge and *oshikundu*) (Tables 2 and 3). However, there was no statistically significant difference in the distribution of CIT among the processed cereals (*P* > 0.05). The recorded maximum concentration was lower than the 11,300 µg/kg reported in flour samples by Angula and co-authors (2024b), but much higher (approximately 8–12 times) than the maximum concentrations reported for baby food in Nigeria (Ojuri et al. 2019; Ayeni et al. 2023). The co-occurrence of FB_1_, a class 2B carcinogen (IARAC 2002), and AFB_1_ in 9.9% as well as in FB_1_, AFB_1_, and CIT in 4.3% of the processed cereal food samples (Fig. 1) suggest additional food safety concerns for the children from possible interactive effects of the toxin mixtures. For example, aflatoxins and fumonisins were reported to contribute to impaired growth in Tanzanian children (Kimanya et al. 2021; Shirima et al. 2015a, b).

Conversely to the findings for aflatoxins, CIT and fumonisins, the contamination of DON and ZEN in ready-to-eat cereal food samples exceeded that of cereal flour samples. Deoxynivalenol was predominantly found in *mahangu* thick/thin porridge with a prevalence of 18.6% and a *mean* ± *SD* of 86.5 ± 75.9 µg/kg, surpassing that of *oshikundu* (9%, 80 ± 25.6 µg/kg) and *mahangu* flour (6%, 70.3 ± 61.3 µg/kg). On the other hand, ZEN was frequently quantified in *oshikundu* samples with *mean* ± *SD* concentrations of 8.4 ± 10.6 µg/kg, higher than *mahangu* thick/thin porridge (2.0 ± 4.1 µg/kg), *omungome* (0.39 µg/kg), sorghum flour (1.65 ± 2.53 µg/kg), and *mahangu* flour (1.0 ± 0.6 µg/kg). The Kruskal–Wallis test also indicated a significant difference in the distribution of DON and ZEN among the processed cereals (*P* < 0.05). Approximately, 2.6 and 0.9% of the ready-to-eat cereal food samples (Table 4) exceeded the 20 µg/kg and 200 µg/kg limits stipulated by the EU for ZEN and DON in children’s food, respectively (EC 2006), indicating food safety issues among consumers. The occurrence of DON and ZEN has also been reported in other studies in Africa, although their concentration levels varied, with ZEN levels generally lower than those found in the current study and DON levels higher (Mahdjoubi et al. 2020; Tshalibe et al. 2020; Ayeni et al. 2023). It is crucial to promote the consumption of a diversified diet among children from Oshana rural households to mitigate the risk of prolonged mycotoxin exposure from contaminated cereals.

### Unregulated metabolites in food samples

Unregulated mycotoxins pose a significant challenge because of their widespread occurrence in foods, particularly in cereals and cereal products (Mihalache et al. 2023). Table 5 depicts the occurrence of unregulated mycotoxins in processed cereal food samples consumed by children from Oshana rural households. Several unregulated metabolites of toxicological importance including MON (range 3.27–724 µg/kg; *mean* ± *SD* 55.7 ± 106), 3-nitropropionic acid (range 0.4–17,200 µg/kg; *mean* ± *SD* 632.4 ± 2272.3), bikaverin (range 0.65–632 µg/kg; *mean* ± *SD* 48.4 ± 105 µg/kg), averufin (range 0.06–101 µg/kg; *mean* ± *SD* 6.2 ± 17.2 µg/kg), rugulusovin (range 0.65–855 µg/kg; *mean* ± *SD* 54.7 µg/kg), tryptophol (range 4.80–8801 µg/kg; *mean* ± *SD* 525 ± 1229 µg/kg), alternariolmethylether (range 0.1–16 µg/kg; *mean* ± *SD* 1.2 ± 2.3 µg/kg), tenuazonic acid (range 7–2419.3 µg/kg; *mean* ± *SD* 110 ± 347.4 µg/kg), and kojic acid (range 25.1–336,954 µg/kg; *mean* ± *SD* 18,717 ± 54,070 µg/kg) were quantified in over 50% of the ready-to-eat cereal foods or cereal flour samples. It is worth noting that, 3-nitropropionic acid, MON, rugulusovin, and tryptophol were found to contaminate more than 60% of both cereal flour and ready-to-eat food samples. The flour samples were quantified with high levels of 3-nitropropionic acid (*median* 14 µg/kg, maximum concentration 17,200 µg/kg) than ready-to-eat food samples (median 16 µg/kg, maximum concentration 2603.4 µg/kg) (Table 5). A similar trend was observed by other authors in Namibia focusing on cereal foods such as pearl millet malt, sorghum malt, and *oshikundu* (Angula et al. 2024b; Nafuka et al. 2019; Misihairabgwi et al. 2018; Misihairabgwi et al. 2018). 3-Nitropropionic acid is known as a neurotoxin (Wang et al. 2017), and its presence in children’s food at elevated concentrations, especially ready-to-eat foods, raises significant health concerns. The co-occurrence of MON with other metabolites such as enniatins and BEAU has been documented (EFSA 2018). Sterigmatocystin, a precursor of aflatoxin which is reported to be teratogenic, mutagenic, and carcinogenic (Gruber-Dorninger et al. 2017; Awuchi et al. 2022) as well as other toxins including MON, alternariolmethylether, and BEAU have been previously reported to contaminate children cereal-based foods in Namibia and other countries (Angula et al. 2024b; Ojuri et al. 2018; Blessa et al. 2012; Juan et al. 2013). The processed cereal foods in this study were found to be contaminated with high levels of unregulated metabolites such as rugulusovin, tryptophol, and kojic acid (Table 5). Although their health impacts on humans have not yet been fully characterized, their presence in food should not be overlooked. Therefore, there is a need for further investigation and the implementation of mitigation strategies to ensure food safety.

**Table 5:** Occurrence of unregulated mycotoxins in processed cereal food samples from Oshana rural households

	**Ready to eat foods *n* = 114**					**Flour samples *n* = 48**				
Metabolite type	*Np*	%*p*	Range	*Median*	*Mean* ± *SD*	*Np*	%*p*	Range	*Median*	*Mean* ± *SD*
Moniliformin	102	89.5	3.3–436.21	15.95	47 ± 3.4	42	87.5	3.3–723.5	14.0	77.1 ± 158.7
Beauvericin	15	13.2	0.22–14.7	0.8	2.36 ± 3.71	10	20.8	0.31–11.62	1.1	3.27 ± 4.42
Beauvericin A	4	3.5	0.09–0.19	0.11	0.12 ± 0.04	2	4.2	0.12–0.62	0.4	0.37 ± 0.4
Enniatin A_1_	1	0.9	0.10–0.10	0.1	0.10 ± 0	0	0	< *LOD*	< *LOD*	< *LOD*
Enniatin B	43	37.7	0.05–1.15	0.15	0.26 ± 0.3	12	25	0.05–0.62	0.13	0.23 ± 0.19
Enniatin B_1_	10	8.8	0.12–0.4	0.19	0.21 ± 0.1	3	6.3	0.11–0.2	0.15	0.15 ± 0.04
Bikaverin	70	61.4	0.65–446.24	8.55	42.7 ± 86.4	24	50	0.70–632.2	16.37	65.08 ± 146.7
Fusaric acid	53	46.5	8.62–4118	108.5	421.95 ± 846.5	11	22.9	13.52–1655.99	200	415.7 ± 506.3
Sterigmatocystin	29	25.4	0.14–3.80	0.47	0.82 ± 0.83	13	27.1	0.13–31.59	2.65	6.54 ± 9.93
Averufin	64	56.1	0.06–33.35	0.38	2.64 ± 6.14	31	64.6	0.06–101.01	0.51	13.51 ± 27.7
3–Nitropropionic acid	73	64	0.43–2600	16.04	156.66 ± 384.05	36	75	0.69–172,000	17.92	1597.04 ± 3768.3
Kojic acid	51	44.7	25.14–41,430	345.62	2969.4 ± 7976	24	50	29.74–336,954	11,477.25	52,180 ± 869,000
Alternariolmethylether	59	51.8	0.11–16	0.41	1.34 ± 2.53	15	31.3	0.13–3.40	15	0.50 ± 0.83
Tenuazonic acid	67	58.8	6.95–2419.3	27.34	129.93 ± 388.6	18	37.5	6.96–237.09	18	36.52 ± 52.98
Emodin	73	64	0.21–34	0.6	2.38 ± 5.71	15	31.3	0.28–21	1.11	5.55 ± 7.85
Rugulusovin	96	84.2	0.65–855.1	14.25	70.40 ± 139.4	35	72.9	0.92–63.72	8.61	11.55 ± 11.74
Tryptophol	106	93	4.80–8800	63.52	701.4 ± 1392	38	79.2	5.62–253.2	15.7	33.00 ± 50.5

Metabolites concentration in µg/kg

*n* number of samples analyzed, *np* number of positive samples, %*p* percent positive samples, *mean* and *median* positive samples only, *SD* standard deviation, < *LOD* less than limit of detection

### Estimated aflatoxin exposure and risk characterization

Table 6 presents the estimated aflatoxin exposure and risk characterization among under 5-year-old children in the Oshana region who consumed ready-to-eat cereal foods. The *APDI* for AFB_1_ and total aflatoxin among the study participants due to high consumption of the two categories of ready-to-eat foods were 0.036 µg/kg bw/day and 0.040 µg/kg bw/day, respectively, based on upper bound scenario. With respect to consumption of individual food types, the *APDI* were at least 2.3 times higher for both AFB_1_ and total aflatoxin from *oshikundu* consumption compared to *mahangu* thick/thin porridge; this was regardless of lower bound or upper bound estimates performed for *mahangu* thick/thin porridge consumption. Correspondingly, the calculated margin of exposure (*MOE*) values among the study participants for both AFB_1_ and total aflatoxin regardless of type of food consumed were all far lesser than 10,000. Notably, the mean exposure levels of the aflatoxins due to the consumption of *oshikundu* and *mahangu* thick/thin porridge in this study was lower than previous exposures reported for children due to consumption of various cereals across Africa (Adetunji et al. 2017; Ojuri et al. 2018; Wielogorska et al. 2019; Ezekiel et al. 2021). As expected, based on the exposure data, the *MOE*s recorded in this study were lowest among children who consumed *oshikundu* (*MOE* 7.6) compared to *mahangu* thick/thin porridge consumers. The *MOE*s observed in the present study were, however, higher than the *MOE*s recorded in other studies from Africa (Adetunji et al. 2017; Wielogorska et al. 2019; Ezekiel et al. 2021). Since the *MOE*s recorded in the present study were below 10,000, a public health risk was considered imminent for these highly vulnerable populations who consume these foods daily (EFSA 2007). Considering the toxicological importance of aflatoxins, addressing its contamination in cereal foods in Sub-Sahara Africa should not be overlooked.

**Table 6:** Aflatoxin exposure and liver cancer risk estimation in under 5-year-old children consuming ready-to-eat foods from Oshana rural households

Food type	Mycotoxin type	Mean mycotoxin concentrations (µg/kg)	*APDI*^c^ (µg/kgbw/day)	*MOE*^d^	Primary liver cancer risk cases^e^ (cancer/year/100,000 people)
*Oshikundu*	AFB_1_ (MB)	1.52	0.053	7.6	0.94
	TAF (MB)	1.59	0.055	7.3	
*Mahangu* thick/thin porridge	AFB1 (LB^a^/UB^b^)	0.51^a^	0.018^a^	22.7^a^	0.31^a^
		0.55^b^	0.018^b^	21.1^b^	0.34^b^
	Total AFs (LB^a^/UB^b^)	0.56^a^	0.019^a^	20.6^a^	
		0.56^b^	0.024^b^	16.8^b^	
All ready-to-eat foods	AFB_1_ (LB^a^/UB^b^	1.02^a^	0.035^a^	11.4^a^	0.63^a^
		1.05^b^	0.036^b^	11.0^b^	0.65^b^
	Total AFs (LB^a^/UB^b^)	1.05^a^	0.036^a^	11.0^a^	
		1.17^b^	0.040^b^	10^b^	

^a^Lower bound

^b^Upper bound and ^MB−^middle bound applied

^c^A *PDI* average probable dietary Intake (µg/kg bw/day for aflatoxins) calculated as: *A PDI=*[mycotoxin concentration (μg/kg) × food comsumption (kg/children/day)]/body weight (kg). The average body weight of children in this study was 12.49 kg, while the average amount of food consumed by children was 0.43 kg

^d^*MOE* margin of exposure for aflatoxin B1 and total aflatoxin was calculated by dividing the benchmark dose lower limit (BMDL10) of 400 ng/kg bw/day) by *APDI* (ng/kg bw/day) (EFSA 2020)

^e^Primary liver cancer risk for aflatoxin B_1_ was estimated by multiplying the AFB1 APDI by the average HCC potency (0.0178 cancers per year per 100,000 per ng AFB_1_ kg^−1^ bw day^−1^)

To further categorize the risk from aflatoxin exposure, the children’s population at risk of liver cancer due to continuous exposure of AFB_1_ (ng/kg bw/day) was calculated, as presented in Table 6. The highest risk of liver cancer was associated with the consumption of *oshikundu* (0.94 cancer cases/year/100,000 population). These risk cancer cases were about 3 times higher than those due to low or high consumption of *mahangu* thick/thin porridge (0.31 and 0.34 cancer cases/year/100,000 population). These results were consistent with those observed in previous studies from Nigeria (Adetunji et al. 2017; Ezekiel et al. 2021). Given the public health concern regarding aflatoxin B_1_ exposure in this study, and the recorded risk of liver cancer among the Oshana rural children, addressing aflatoxin contamination in cereal food should be a high priority for risk management action. Aflatoxins are carcinogenic and genotoxic even at low levels of exposure; therefore, minimizing their presence in foods and exposure levels is crucial (IPCS 2020). Consequently, there is a pressing need for mitigation strategies, including the improvement of grain storage facilities and enhancing food preparation and best storage practices among rural households in Namibia.

### Estimated mycotoxin exposure and risk characterization to non-genotoxin and non-carcinogenic mycotoxins

Table 7 presents the average *PDI* and %*TDI* levels of mycotoxin exposure for Oshana rural children, revealing notable exposure risks to certain mycotoxins. Children from Oshana rural households recorded a worrisome level of exposure to FB_1_ and total fumonisins through the consumption of *mahangu* thick/thin porridge, with a high *APDI* of 1.96 µg/kg bw/day and 3.31 µg/kg bw/day, respectively, along with corresponding high %*TDI* of 196% and 331.8%. The %*TDI* of FB_1_ and total fumonisin due to *mahangu* thick/thin porridge consumption among the rural children was slightly higher than for *oshikundu* (114 and 217.5%, respectively). These levels substantially surpassed the 1 µg/kg bw/day limit established by EFSA (2018) by several hundred folds. Fumonisins exposure results in this study were 2.4–5.5 times lower than those reported among children in other African countries due to the consumption of mycotoxin-contaminated cereals (Kimanya et al. 2014; Shepard et al. 2007; Ojuri et al. 2018; Ezekiel et al. 2021). Chronic childhood exposure to dietary fumonisins in the present study suggests a risk of stunted growth, development of esophageal cancer, and neural tube defects at the later stage of development (Missmer et al. 2006; Shirima et al. 2015a, b).

**Table 7:** Exposure estimation and risk characterization of non-genotoxic and non-carcinogenic mycotoxins among under 5 years children from Oshana region

Food type	Mycotoxin type	Fumonisin B_1_		Total fumonisins		Zearalenone		Deoxynivalenol		Citrinin		Beauvericin		Moniliformin
*Oshikundu*		LB	UB	LB	UB	LB	UB	LB	UB	LB	UB	LB	UB	MB
		31.3	32.9	53.44	62.88	2.87	2.92	7.14	8.21	50.07	50.16	0.49	0.51	65.05
	*APDI* (µg/kgbw/day)	1.08	1.14	1.85	2.18	0.099	0.101	0.247	0.280	1.732	1.735	0.017	0.018	2.250
	%*TDI*^a^/*MOE*^b^	108^a^	114^a^	185	218	39.7^a^	40.4^a^	24.7^a^	28.4^a^	0.12^b^	0.12^b^	5301^b^	5096^b^	88.9^b^
*Mahangu* thick/thin porridge	Mean mycotoxin concentration (µg/kg)	50	56.5	82.1	96	0.55	0.61	16.0	17	22.3	22.43	0.15	0.17	24.5
	*APDI* (µg/kgbw/day)	1.73	1.96	2.84	3.31	0.019	0.021	0.55	0.59	0.77	0.78	0.005	0.006	0.85
	%*TDI*^a^/*MOE*^b^	173^a^	196^a^	284.1	331	7.67^a^	8.4^a^	53.85^a^	58.68^a^	0.26^b^	0.26^b^	17665^b^	15390^b^	236^b^
All ready-to-eat food samples	Mean mycotoxin concentration (µg/kg)	43	44.5	70.0	79.1	1.73	1.78	11.3	12.5	36.5	36.5	0.32	0.34	44.2
	*APDI* (µg/kgbw/day)	1.49	1.54	2.42	2.74	0.0599	0.061	0.39	0.43	1.26	1.264	0.011	0.012	1.53
	%*TDI*^a^/*MOE*^b^	149^a^	154^a^	242^a^	274^a^	24^a^	25^a^	39.2^a^	43.3^a^	0.16^b^	0.16^b^	8076^b^	7665^b^	131^b^

*LB* lower bound, *UB* upper bound, *MB* middle bound, *APDI* average probable dietary intake (µg/kg bw/day for aflatoxins) calculated as: *A PDI=*[mycotoxin concentration (μg/kg) × food comsumption (kg/children/day)]/body weight (kg). The average body weight of children in this study was 12.49 kg, while the average amount of food consumed by children was 0.43 kg

^a^%*TDI* percentage tolerable daily intake-calculated by dividing *APDI* by tolerable daily intake. A *TDI* of 1 µg/kg bw/day was adopted for fumonisins and deoxynivalenol (JECFA 2017) and 0.25 µg/kg bw/day for zearalenone (EFSA 2012)

^b^*MOE* margin of exposure was calculated by dividing the reference point by *APDI*. Reference points: citrinin, the level of no concern for nephrotoxicity (0.2 μg/kg bw/day) (EFSA 2012), beauvericin, the lowest dose of 90 μg/kg bw/day and moniliformin, benchmark dose lower limit (BMDL05) of 200 μg/kg bw/day (EFSA 2012, 2014, 2018)

Furthermore, the high consumption of both types of ready-to-eat-foods also exposed children to DON, ZEN, CIT, BEAU, and MON. The *APDI* and %*TDI*/*MOE* were as follows: DON (*APDI* 0.43 µg/kg bw/day; %*TDI* 43.3%), ZEN (*APDI* 0.061 µg/kg bw/day; %*TDI* 24.6%), CIT (*APDI* 1.26 µg/kg bw/day; *MOE* 0.16), *BEA* (*APDI* 0.012 µg/kg bw/day; *MOE* 7665) and MON (*APDI* 1.53 µg/kg bw/day; *MOE* 130.8) (Table 7). Considering the ready-to-eat food categories, exposure to ZEA, CIT, BEA, and MON were generally higher from the consumption of *oshikundu* compared to *mahangu* thick or thin porridge consumption, except for deoxynivalenol exposure which was mostly due to consumption of *mahangu* thick/thin porridge (Table 7). The mean exposure levels to DON and ZEN were below the *TDI* values set by EFSA (1 µg/kg bw/day for DON and 0.25 µg/kg bw/day for ZEN) (EFSA (2014, 2018); however, their presence in foods still warrant attention. The *APDI* for DON and ZEN exposure recorded in this study was lower than the levels reported among children from other African countries (Adetunji et al. 2017; Mahdjoubi et al. 2020; Tshalibe et al. 2020). Chronic exposure of children to zearalenone and deoxynivalenol has long-term consequences, including estrogenic effects and inhibition of proteosynthesis and oxidative stress (Lee and Ryu 2015; Ferrigo et al. 2016; Haus et al. 2021). Therefore, the presence of zearalenone and deoxynivalenol in cereal foods, especially ready-to-eat foods intended for children, is a health concern, necessitating mitigating strategies.

Moreover, the *APDI* and MOEs to CIT, BEAU, and MON in the current study (Table 7) surpassed those reported by previous researchers (Ojuri et al. 2019; Ali et al. 2020; Ezekiel et al. 2021). The *APDI* for CIT among rural children in this study exceeded the EU reference point of 0.2 µg/kg bw/day, indicating a level of concern for nephrotoxicity. Additionally, the low *MOE* values for MON and the co-exposure to MON and BEAU suggest potential health concerns, despite the lack of clear conclusions regarding the toxicity and exposure to these mycotoxins (EFSA 2018). The co-exposure of children to different types of mycotoxins due to consumption of cereal foods observed in this study, including the emerging mycotoxins, may lead to adverse health effects due to mycotoxins synergistic effects (Clarke et al. 2014; Klarić et al. 2012; Stoev et al. 2009). Co-occurrence of aflatoxins, fumonisins, and citrinin in this vulnerable population, with their consequent exposure values, indicates a potential health risk of stunting and liver cancer in the children (Shirima et al. 2015a, b; Kimanya et al. 2021; Wu et al. 2024). Although previous studies in the Oshana and Oshikoto regions of Namibia have revealed a high prevalence of stunting among children consuming staple cereals (Angula et al. 2024a), there is a dearth of research linking impaired growth to mycotoxin exposure, necessitating further investigation.

## Conclusion

Processed cereal foods consumed by under 5-year-old children from rural households in Oshana region revealed the presence of major mycotoxins, with aflatoxin, fumonisin, zearalenone, deoxynivalenol, and citrinin being the most prevalent. Notably, sorghum flour showed higher levels of AFB_1_ compared to other processed cereal food types. Statistically, there was a significant difference in the distribution of AFB_1_, FB_1_, DON, and ZEN among the processed cereal foods. Alarmingly, a considerable portion of the food samples exceeded the EU limits for aflatoxin B_1_ and fumonisin B_1_ in children’s foods, indicating potential food safety concerns associated with mycotoxin exposure among children. The quantification of emerging mycotoxins like moniliformin, bikaverin, tenuazonic acid, averufin, and alternariolmethylether in over 50% of the samples underscores a potential food safety concern among the cereal food consumers. Furthermore, the presence of 3-nitropropionic acid, at high levels in over 70% of the food samples, especially in sorghum flour, warrants immediate attention to mitigate potential health risks. Exposure to AFB_1_ indicated a public health risk among children, and risk of liver cancer was frequently observed among children who consumed *oshikundu* than *mahangu* thick/thin porridge. The %*TDI* for fumonisin was exceeded, indicating health risks such as impaired growth among children, while the recorded low *MOE* to citrinin indicated a a health concern to nephrotoxicity among the rural children. The co-occurrence of aflatoxins and fumonisins and citrinin in this vulnerable population, with their consequent (co-)exposure values, indicated a potential health risk to children. The study therefore highlights the urgent need for interventions to mitigate mycotoxin contamination in staple foods; these should include best grain planting, harvest and storage practices, as well as appropriate postharvest food handling and preparation practices to safeguard the health of the rural children in Namibia. A diversified diet is also recommended among the Oshana rural children to mitigate the risk of chronic mycotoxin exposure through consumption of cereal foods.

## Supplementary Information

Below is the link to the electronic supplementary material.

Supplementary file1 (DOCX 49 KB)

Supplementary file2 (DOCX 17 KB)

## Data Availability

"The data analysed during this study are available from the corresponding author upon request".

## References

[CR1] Abdulrazzaq YM, Osman N, Yousif ZM, Trad O (2004) Morbidity in neonates of mothers who have ingested aflatoxins. Ann Trop Paediatr 24:145–151. 10.1179/027249304225013420 15186543 10.1179/027249304225013420

[CR2] Adetunji MC, Atanda OO, Ezekiel CN (2017) Risk assessment of mycotoxins in stored maize grains consumed by infants and young children in Nigeria. Child (Bas) 4(7):58. 10.3390/children4070058 10.3390/children4070058 PMC5532550 28698507

[CR3] Ali N (2020) Degen GH (2020) Biological monitoring for ochratoxin A and citrinin and their metabolites in urine samples of infants and children in Bangladesh. Myc Res 36:409–417. 10.1007/s12550-020-00407-7 10.1007/s12550-020-00407-7 32820428

[CR4] Alvito P, Pereira-da-Silva L (2022) Mycotoxin Exposure during the first 1000 days of life and its impact on children’s health: a clinical overview. Toxins 14(3):189. 10.3390/toxins14030189 35324686 10.3390/toxins14030189 PMC8955462

[CR5] Angula M, Ishola A, Tjiurutue M et al (2024a) Association of food consumption patterns and nutritional status of children under 5 years from rural households in Northern regions. Namibia BMC Nutr 10(1):51. 10.1186/s40795-024-00833-1 38500224 10.1186/s40795-024-00833-1 PMC10949813

[CR6] Angula M, Ishola A, Tjiurutue M et al (2024b) Mycotoxins in stored cereals from rural households in central northern Namibia. F Cont. 10.1016/j.foodcont.2024.110532

[CR7] Assuncao R, Vasco E, Nunes B, Loureiro S, Martins C, Alvito P (2015) Single-compound and cumulative risk assessment of mycotoxins present in breakfast cereals consumed by children from Lisbon Region, Portugal. F Chem Toxicol 86:274–281 10.1016/j.fct.2015.10.017 26545619

[CR8] Awala SK, Hove K, Wanga MA et al (2019) Rainfall trend and variability in semi-arid northern Namibia: implications for smallholder agricultural production. Welw Int Jour of Agric Scie 1:1–25. 10.32642/wijas.v1i0.1364

[CR9] Awuchi CG, Ondari EN, Nwozo S et al (2022) Mycotoxins’ toxicological mechanisms involving humans, livestock and their associated health concerns: a review. Tox 14:167. 10.3390/toxins14030167 10.3390/toxins14030167 PMC8949390 35324664

[CR10] Ayeni KI, Sulyok M, Krska R et al (2023) Mycotoxins in complementary foods consumed by infants and young children within the first 18 months of life. F Cont 144:1–10

[CR11] Blessa J, Marín R, Lino CM, Mañes J (2012) Evaluation of enniatins A, A1, B, B1 and beauvericin in Portuguese cereal-based foods. F Addit Contam 29:1727–1735 10.1080/19440049.2012.702929 22845312

[CR12] CAST-Council for Agricultural Science and Technology (2003) Mycotoxins: risks in plant, animal and human systems. Task Force Report no. 139. Cou Agr Scie Techn Ames IA 1–191

[CR13] Chen C, Riley RT, Wu F (2018) Dietary fumonisin and growth impairment in children and animals: a review. Comp Rev F Scie and F Saf 17:1448–1464 10.1111/1541-4337.12392 33350142

[CR14] Chilaka AC, Mally A (2020) Mycotoxin occurrence, exposure and health implications in infants and young children in Sub-Saharan Africa: a review. Foods 9(11):1585. 10.3390/foods9111585 10.3390/foods9111585 PMC7693847 33139646

[CR15] Chuisseu PDD, Abia WA, Zibi B et al (2018) Safety of breast milk vis-a-vis common infant formula and complementary foods from western and centre regions of Cameroon from mycotoxin perspective. Rec Adv Food Scie 1:23–31

[CR16] Clarke R, Connolly L, Frizzell C, Elliott CT (2014) Cytotoxic assessment of the regulated, co-existing mycotoxins aflatoxin B1, fumonisin B1 and ochratoxin, in single, binary and tertiary mixtures. Toxic 90:70–81 10.1016/j.toxicon.2014.07.019 25110174

[CR17] Codex Alimentarius Commission (1995) General standard for contaminants and toxins in food and feed (Codex STAN 193–1995). Food and Agriculture Organization of the United Nations (FAO) and World Health Organization (WHO). Available from: https://www.fao.org/fileadmin/user_upload/agns/pdf/CXS_193e.pdf

[CR18] Doughari JH (2015) The occurrence, properties and significance of citrinin mycotoxin. J Plan Pathol Microbiol 6:11

[CR19] EC – European Commission (2002) 2002/657/EC: commission decision of 12 August 2002 implementing council directive 96/23/EC concerning the performance of analytical methods and the interpretation of results (Text with EEA relevance) (notified under document number C(2002) 3044). Off J 221:8–36. http://data.europa.eu/eli/dec/2002/657/oj

[CR20] EC – European Commission (2006) Commission regulation (EC) No 1881/2006 of 19 December 2006 setting maximum levels for certain contaminants in foodstuffs. Off J Eur Union L 364:5–24 Last consolidated version available from: https://eur-lex.europa.eu/legal-content/DE/AUTO/?uri=CELEX:02006R1881-20180319

[CR21] EFSA- European Food Safety Authority (2007) Opinion of the Scientific Panel on Contaminants in the Food Chain on a request from the commission related to the potential increase of consumer health risk by a possible increase of the existing maximum levels for a flatoxins in almonds, hazel nuts and pistachios and derived products. EFSA J 446:1 –127. https://efsa.onlinelibrary.wiley.com/journal/ 18314732

[CR22] EFSA-European Food Safety Authority (2010) Management of left-censored data in dietary exposure assessment of chemical substances. EFSA J 8:1557

[CR23] EFSA-European Food Safety Authority (2012) Scientific opinion on the risks for public and animal health related to the presence of citrinin in food and feed. EFSA J 10(3):1–82. 10.2903/j.efsa.2012.2605 10.2903/j.efsa.2012.2605 PMC13151811 42111980

[CR24] EFSA-European Food Safety Authority (2020) Scientific opinion on the risk assessment of aflatoxins in food. The EFSA Journal 18:6040. 10.2903/j.efsa.2020.6040 10.2903/j.efsa.2020.6040 PMC7447885 32874256

[CR25] EFSA-European Food Safety Authority (2014) Scientific opinion on the risks to human and animal health related to the presence of beauvericin and enniatins in food and feed. EFSA J 12(8). 10.2903/j.efsa.2014.3802

[CR26] EFSA-European Food Safety Authority (2018) Risks to human and animal health related to the presence of moniliformin in food and feed. EFSA Journal, 16(3). 10.2903/j.efsa.2018.5082 10.2903/j.efsa.2018.5082 PMC7009678 32625822

[CR27] Ezekiel CN, Sulyok M, Ogara IM et al (2019) Mycotoxins in uncooked and plate-ready household food from rural northern Nigeria. F Chem Toxicol 128:171–179. 10.1016/j.fct.2019.04.002 10.1016/j.fct.2019.04.002 30965105

[CR28] Ezekiel CN, Ayeni KI, Akinyemi MO et al (2021) Dietary risk assessment and consumer awareness of mycotoxins among household consumers of cereals, nuts and legumes in North-Central Nigeria. Tox 13(9):635. 10.3390/toxins13090635 10.3390/toxins13090635 PMC8472633 34564639

[CR29] FAO/WHO-Food and Agriculture /World Health Organization (1998) Joint FAO/WHO expert committee on food additives – evaluation of certain food additives and contaminants: forty-ninth report of the joint FAO/WHO expert committee on food additives. https://iris.who.int/handle/10665/42142 10079756

[CR30] Ferrigo D, Raiola A, Causin R (2016) Fusarium toxins in cereals: occurrence, legislation, factors promoting the appearance and their management. Mol 21:627 10.3390/molecules21050627 PMC6274039 27187340

[CR31] Foerster C, Monsalve L, Ríos-Gajardo G (2022) Mycotoxin exposure in children through breakfast cereal consumption in Chile. Tox 14(5):324. 10.3390/toxins14050324 10.3390/toxins14050324 PMC9146524 35622571

[CR32] Gong YY, Watson S, Routledge MN (2016) Aflatoxin exposure and associated human health effects: a review of epidemiological studies. F Saf 4:14–27 10.14252/foodsafetyfscj.2015026 PMC6989156 32231900

[CR33] Gruber-Dorninger C, Novak B, Nagl V, Berthiller F (2017) Emerging mycotoxins: beyond traditionally determined food contaminants. J Agric and F Chem 65(33):7052–7070. 10.1021/acs.jafc.6b03413 10.1021/acs.jafc.6b03413 27599910

[CR34] Haus M, Žatko D, Vašková J et al (2021) The effect of humic acid in chronic deoxynivalenol intoxication. Environ Sci Pollut Res 28:1612–1618. 10.1007/s11356-020-10581-x 10.1007/s11356-020-10581-x 32851525

[CR35] IARC-International Agency for Research on Cancer (2002) Some traditional herbal medicines, some mycotoxins, naphthalene, and stryene. IARC Monogr Eval Carcinog Risks Hum 82:171–300 PMC4781602 12687954

[CR36] IPCS-International Programme on Chemical Safety /GEMS (1995) Food Euro workshop on reliable evaluation of low-level contamination of food 5. WHO, Kulmbach, Germany

[CR37] IPCS-International Programme on Chemical Safety (2020) Chapter 6: dietary exposure assessment for chemicals in food. https://www.who.int/docs/defaultsource/chemical-safety/ehc240-chapter6-edited(4-1).pdf?sfvrsn=96810319_0

[CR38] Iqbal SZ, Rabbani T, Asi MR, Jinap S (2014) Assessment of aflatoxins, ochratoxin A and zearalenone in breakfast cereals. F Chem 157:257–262 10.1016/j.foodchem.2014.01.129 24679779

[CR39] JECFA- Joint FAO/WHO Expert Committee on Food Additives (2017) Evaluation of certain mycotoxins in food: eighty-third report of the joint FAO/WHO expert committee on food additives. In WHO Technical Report Series No 1002, WHO: Geneva, Switzerland. Available online:https://apps.who.int/iris/bitstream/handle/10665/254893/9789241210027eng.pdf?sequence=1. Accessed 05 July 2024.

[CR40] Juan C, Mañes J, Raiola A, Ritieni A (2013) Evaluation of beauvericin and enniatins in Italian cereal products and multi-cereal food by liquid chromatography coupled to triple quadrupole mass spectrometry. F Chem 140(4):755–762 10.1016/j.foodchem.2012.08.021 23692763

[CR41] Kaela CR, Lilly M, Rheeder JP et al (2023) Mycological and multiple mycotoxin surveillance of sorghum and pearl millet produced by smallholder farmers in Namibia. Curr Microb 80(5):164. 10.1007/s00284-023-03263-7 10.1007/s00284-023-03263-7 PMC10073170 37014446

[CR42] Kamala A, Kimanya M, Lachat C et al (2017) Risk of exposure to multiple mycotoxins from maize-based complementary foods in Tanzania. J Agr Food Chem 65(33):7106–7114. 10.1021/acs.jafc.6b03429 28830150 10.1021/acs.jafc.6b03429

[CR43] Kimanya ME, Shirima CP, Magoha H et al (2014) Co-exposures of aflatoxins with deoxynivalenol and fumonisins from maize based complementary foods in Rombo. Northern Tanzania F Cont 41:76–81. 10.1016/j.foodcont.2013.12.034

[CR44] Kimanya ME, Routledge MN, Mpolya E et al (2021) Estimating the risk of aflatoxin-induced liver cancer in Tanzania based on biomarker data PLOS One, 1–11 10.1371/journal.pone.0247281 10.1371/journal.pone.0247281 PMC7951873 33705417

[CR45] Klarić MS, Zeljezic D, Rumora L et al (2012) A potential role of calcium in apoptosis and aberrant chromatin forms in porcine kidney PK15 cells induced by individual and combined ochratoxin A and citrinin. Arch of Tox 86:97–107 10.1007/s00204-011-0735-9 21739216

[CR46] Kowalska K, Habrowska-Górczynska DE, Piastowska-Ciesielska AW (2016) Zearalenone as an endocrine disruptor in humans. Environ Toxicol Pharmacol 48:141–149 10.1016/j.etap.2016.10.015 27771507

[CR47] Lee HJ, Ryu D (2015) Advances in mycotoxin research: public health perspectives. J Food Sci 80:T2970–T2983. 10.1111/1750-3841.13156 26565730 10.1111/1750-3841.13156

[CR48] Lombard MJ (2014) Mycotoxin exposure and infant and young child growth in Africa: what do we know? Ann Nutr Metab 64:42–52 25341872 10.1159/000365126

[CR49] Magnusson B, Ornemark U (2014) Eurachem guide: the fitness for purpose of analytical methods. A laboratory guide to method validation and related topics. www.eurachem.org

[CR50] Mahdjoubi CK, Arroyo-Manzanares N, Hamini-Kadar N et al (2020) Multi-mycotoxin occurrence and exposure assessment approach in foodstuffs from Algeria. Tox 12:194. 10.3390/toxins12030194 10.3390/toxins12030194 PMC7150886 32204439

[CR51] Malachova A, Sulyok M, Beltran et al (2014) Optimization and validation of a quantitative liquid chromatography-tandem mass spectrometric method covering 295 bacterial and fungal metabolites including all regulated mycotoxins in four model food matrices. J chromat 1362:145–156. 10.1016/j.chroma.2014.08.037 10.1016/j.chroma.2014.08.037 25175039

[CR52] Mhata P, Rennie TW, Small LF et al (2017) Distribution of hepatitis B virus infection in Namibia. S Afr Med 107(10):882–886. 10.7196/SAMJ.2017.v107i10.12171 10.7196/SAMJ.2017.v107i10.12171 29022533

[CR53] Mihalache OA, De Boevre M, Dellafiora L et al (2023) The occurrence of non-regulated mycotoxins in foods: a systematic review. Tox 15(9):583. 10.3390/toxins15090583 10.3390/toxins15090583 PMC10534703 37756008

[CR54] Misihairabgwi JM, Ishola A, Quaye I et al (2018) Diversity and fate of fungal metabolites during the preparation of oshikundu, a Namibian traditional fermented beverage. W Mycotox J 11(3):471–481

[CR55] Misihairabgwi J, Angula M, Sivhute E et al (2023) Chapter 13 Natural food toxicants and health implications. In OA Ijabadeniyi and OF Olagunju (Eds.), Food safety and toxicology, De Gruyter. 10.1515/9783110748345-013

[CR56] Missmer SA, Suarez L, Felkner M et al (2006) Exposure to fumonisins and the occurrence of neural tube defects along the Texas-Mexico border. Environ Heal Pers 114:237–241 10.1289/ehp.8221 PMC1367837 16451860

[CR57] Nafuka SN, Misihairabgwi JM, Bock R et al (2019) Variation of fungal metabolites in sorghum malts used to prepare Namibian traditional fermented beverages Omalodu and Otombo. Tox 11:165. 10.3390/toxins110301651-18 10.3390/toxins11030165 PMC6468557 30884826

[CR58] Naing L, Winn T, Rusli B (2006) Practical issues in calculating the sample size for prevalence studies. Arch of Orofac Scie 1:9–14

[CR59] Namibia population and housing census (2011) Namibia 2011 Population and housing census main report. https://cms.my.na/assets/documents/p19dmn58guram30ttun89rdrp1.pdf

[CR60] Ojuri OT, Ezekiel CN, Sulyok M et al (2018) Assessing the mycotoxicological risk from consumption of complementary foods by infants and young children in Nigeria. F Chem Toxic 121:37–50. 10.1016/j.fct.2018.08.025 10.1016/j.fct.2018.08.025 30118820

[CR61] Ojuri OT, Ezekiel CN, Eskola MK et al (2019) Mycotoxin co-exposures in infants and young children consuming household and industrially processed complementary foods in Nigeria and risk management advice. F Cont 98:312–322. 10.1016/j.foodcont.2018.11.049

[CR62] Oshana regional council (2023) Oshana regional profile. https://oshanarc.gov.na/

[CR63] Peraica M, Richter D, Rasic D (2014) Mycotoxicoses in children. Arch Ind Hyg Toxicol 65:347–363 10.2478/10004-1254-65-2014-2557 25720023

[CR64] Shephard GS, Marasas WFO, Burger HM et al (2007) Exposure assessment for fumonisins in the former Transkei region of South Africa. F Addit and Contam 24:621–629 10.1080/02652030601101136 17487603

[CR65] Shirima CP, Kimanya ME, Routledge MN et al (2015a) A prospective study of growth and biomarkers of exposure to aflatoxin and fumonisin during early childhood in Tanzania. Environ Heal Persp 123:173–178 10.1289/ehp.1408097 PMC4314247 25325363

[CR66] Shirima CP, Kimanya ME, Routledge MN et al (2015b) A prospective study of growth and biomarkers of exposure to aflatoxin and fumonisin during early childhood in Tanzania. Env Heal Persp 123(2):173–178. 10.1289/ehp.1408097 10.1289/ehp.1408097 PMC4314247 25325363

[CR67] Stadler D, Sulyok M, Berthiller F, Schuhmacher R, Krska R (2018) The contribution of lot-to-lot variation to the measurement uncertainty of an LC-MS-based multi-mycotoxin assay. Anal Bioanal Chem 410:4409–4418. 10.1007/s00216-018-1096-5 10.1007/s00216-018-1096-5 PMC6021480 29713754

[CR68] Stoev SD, Denev S, Dutton M, Nkosi B (2009) Cytotoxic effect of some mycotoxins and their combinations on human peripheral blood mononuclear cells as measured by MTT assay. The Op Toxin J 2:1–8

[CR69] Sulyok M, Stadler D, Steiner D, Krska R (2020) Validation of an LC-MS/MS-based dilute-and-shoot approach for the quantification of >500 mycotoxins and other secondary metabolites in food crops: challenges and solutions. Anal Bioan Chem 412(11):2607–2620 10.1007/s00216-020-02489-9 PMC7136310 32078002

[CR70] Sulyok M, Suman M, Krska R (2024) Quantification of 700 mycotoxins and other secondary metabolites of fungi and plants in grain products. npj Sci Food 8:49. 10.1038/s41538-024-00294-7 10.1038/s41538-024-00294-7 PMC11297916 39097644

[CR71] Tshalibe RS, Rheeder JP, Alberts JF et al (2020) Multi-mycotoxin exposure of children (0–24 months) in rural maize-subsistence farming areas of the Eastern Cape Province. South Africa W Myc J 13(3):401–410. 10.3920/WMJ2019.2439

[CR72] Wang L, Wang J, Yang L et al (2017) Effect of Praeruptorin C on 3-nitropropionic acid induced Huntington’s disease-like symptoms in mice. Biomed Pharmac 86:81–87 10.1016/j.biopha.2016.11.111 27939523

[CR73] Waseem A, Shoaib AS, Ashif S et al (2015) Human exposure to mycotoxins: a retrospective review of leading toxins and metabolites in human biological matrices. J Chem Soc Pak 36:1196–1214

[CR74] Wielogorska E, Mooney M, Eskola M et al (2019) Occurrence and human-health impacts of mycotoxins in Somalia. J Agr Food Chem 67(7):2052–2060. 10.1021/acs.jafc.8b05141 30694057 10.1021/acs.jafc.8b05141

[CR75] Wu H, Xu Y, Gong YY et al (2024) Effects of aflatoxin and fumonisin on gene expression of growth factors and inflammation-related genes in a human hepatocyte cell line. Mut 39(3):181–195. 10.1093/mutage/geae005 10.1093/mutage/geae005 PMC11040159 38468450

