# Supplementary material for: Mycotoxin exposure through the consumption of processed cereal food for children (< 5 years old) from rural households of Oshana, a region of Namibia
Source: Mycotoxin Res. 2025 Jan 14;41(1):249–65. doi: 10.1007/s12550-024-00580-z (PMC11759469; doi:10.1007/s12550-024-00580-z)
Supplement: Supplementary file 1 — Supplementary file1 (DOCX 49 KB) [file 12550_2024_580_MOESM1_ESM.docx]

**Table S1: Occurrence of other secondary metabolites in processed cereal food samples from Oshana region.**

| **Ready to eat foods (n=114)** | | | | | **Flour samples (n=48)** | | | |
| --- | --- | --- | --- | --- | --- | --- | --- | --- |
| Metabolite type | Np | %p | Range | Mean±SD | Np | %p | Range | Mean±SD |
| Acuminatum B | 7 | 6.1 | 27.67-405.4 | 164.28±156 | 4 | 8.3 | 20-271.44 | 141.85±103.61 |
| Acuminatum C | 3 | 2.6 | 94.08-131.3 | 108.46±20 | 3 | 6.3 | 26.32-90.40 | 53.43±33.2 |
| Antibiotic Y | 4 | 3.5 | 1.23-5.72 | 2.60±2.10 | 6 | 12.5 | 0.67-8.19 | 2.60±2.83 |
| Aurofusarin | 55 | 48.2 | 2.05-698.1 | 52.35±116.8 | 22 | 45.8 | 2.49-6151 | 639.68±1498 |
| Butenolid | 2 | 1.8 | 30.23-82.7 | 56.45±37.1 | 6 | 12.5 | 35.42-804.8 | 192.62±302 |
| Deoxyfusapyron | 5 | 4.4 | 3.43-18.42 | 12.86±6.77 | 2 | 4.2 | 8.88-89.24 | 49.06±56.82 |
| Epiequisetin | 39 | 34.2 | 0.16-205.7 | 16.66±42.54 | 5 | 10.4 | 0.16-2.90 | 1.29±1.29 |
| Equisetin | 49 | 43 | 0.34-624.53 | 36.42±105.63 | 13 | 27.1 | 0.69-206.5 | 34.74±66.5 |
| Fusaproliferin | 4 | 3.5 | 14.73-248.3 | 83.08±111.26 | 1 | 2.1 | 79.96-79.96 | 79.96± |
| Fusapyron | 3 | 2.6 | 1.13-2.25 | 1.55±0.61 | 3 | 6.3 | 0.71-3.93 | 2.76±1.78 |
| Fusarin C | 0 | 0 | <LOD | <LOD | 5 | 10.4 | 137.16-401.45 | 202.04±112.15 |
| Sambutoxin | 4 | 3.5 | 0.15-0.22 | 0.19±0.03 | 1 | 2.1 | 0.07-0.07 | 0.07±0 |
| Siccanol | 4 | 3.5 | 129.90-261.4 | 176.50±61.50 | 0 | 0 | <LOD | <LOD |
| W493 | 41 | 36 | 2.98-417.2 | 52.90±105.6 | 12 | 25 | 4.06-180.3 | 38.50±52.5 |
| O-Methyl | 6 | 5.3 | 0.22-3.94 | 1.04±1.45 | 9 | 18.75 | 0.16-1.65 | 0.72±0.51 |
| Versicolorin A | 7 | 6.1 | 0.4-1.0 | 0.67±0.25 | 10 | 20.83 | 0.46-16.13 | 2.75±5 |
| Versicolorin C | 14 | 12.3 | 0.03-1.00 | 0.34±0.31 | 12 | 25 | 0.12-5.73 | 1.24±1.72 |
| Versiconal Acetat | 0 | 0 | <LOD | <LOD | 1 | 2.08 | 0.23-0.23 | 0.23±0 |
| Averantin | 30 | 26.3 | 0.10-13.48 | 1.76±2.55 | 18 | 37.5 | 0.12-116.13 | 19.79±36.98 |
| Norsolorinic acid | 9 | 7.9 | 0.81-5.41 | 1.86±1.45 | 13 | 27.08 | 0.99-53.52 | 11.23±15.75 |
| seco-Sterigmatocystin | 2 | 1.8 | 0.17-0.17 | 0.17±0.00 | 3 | 6.25 | 0.33-3.14 | 1.29±1.61 |
| Aspertoxin | 4 | 3.5 | 12925-171100 | 69194±70405 | 4 | 8.33 | 15660-52520 | 27830±17035 |
| Hydroxy | 0 | 0 | <LOD | <LOD | 5 | 10.42 | 16648-63920 | 36074±17181 |
| Bis(methylthio)gliotoxin | 7 | 6.1 | 2.82-20.6 | 12.10±7.4 | 8 | 16.67 | 2.24-361.92 | 60.88±123 |
| Festuclavine | 24 | 21.1 | 0.06-338.5 | 31.12±92.3 | 12 | 25 | 0.14-50.6 | 7.81±16.34 |
| Fumagillin | 1 | 0.9 | 40.52-40.52 | 40.52±0 | 4 | 8.33 | 6.48-221.44 | 70.21±101.36 |
| Fumigaclavine A | 8 | 7 | 0.33-3.95 | 1.34±1.28 | 8 | 16.67 | 0.88-51.69 | 12.80±17.82 |
| Fumigaclavine C | 12 | 10.5 | 1.16-42.1 | 12.67±13.26 | 9 | 18.75 | 5.01-792 | 153.85±252.4 |
| Fumiquinazolin A | 5 | 4.4 | 1.43-13.13 | 7.12±5.65 | 7 | 14.58 | 0.71-74.8 | 20.42±28.2 |
| Fumiquinazolin D | 12 | 10.5 | 2.31-70.1 | 17.85±22.6 | 9 | 18.75 | 4.75-528.5 | 138.78±209.8 |
| Fumitremorgin B | 0 | 0 | <LOD | <LOD | 1 | 2.08 | 3.55-3.55 | 3.55±0 |
| Fumitremorgin C | 3 | 2.6 | 1.46-3.3 | 2.70±1.1 | 4 | 8.33 | 1.93-27.35 | 9.31±12.08 |
| Gliotoxin | 0 | 0 | <LOD | <LOD | 2 | 4.17 | 19.22-19.41 | 19.31±0.14 |
| Helvolic acid | 6 | 5.3 | 2.65-126.7 | 53±48.3 | 7 | 14.58 | 11.86-1064 | 233.23±399 |
| Helvolinic acid | 2 | 1.8 | 10.59-11.93 | 11.26±0.95 | 3 | 6.25 | 9.12-74.92 | 36.71±34.16 |
| Pseurotin A | 5 | 4.4 | 4.74-27.8 | 13.43±8.83 | 6 | 12.5 | 6.20-117.3 | 28.24±44 |
| Cytochalasin E | 4 | 3.5 | 2.08-5.31 | 4.19±1.43 | 3 | 6.25 | 2.89-26.51 | 13.00±12.2 |
| Deoxynortryptoquivalin | 1 | 0.9 | 3.49-3.5 | 3.49±0 | 3 | 6.25 | 2.82-4.04 | 3.34±0.63 |
| Deoxytryptoquivaline A | 2 | 1.8 | 4.41-15.6 | 10.02±7.9 | 1 | 2.1 | 13.80-13.80 | 13.80±0 |
| Tryptoquivaline A | 1 | 0.9 | 2.12-2.1 | 2.12±0 | 1 | 2.1 | 5.98-5.98 | 5.98±0 |
| Tryptoquivaline F | 6 | 5.3 | 2.09-75580 | 23048±35917 | 6 | 12.5 | 10.47-33440 | 5602.83±13637 |
| Asperfuran | 7 | 6.1 | 43.22-198.57 | 82.52±52.66 | 12 | 25 | 30.01-4858.82 | 803.07±1360.03 |
| Aspergillimide | 1 | 0.9 | 0.50-0.50 | 0.50±0 | 3 | 6.3 | 1.23-1.78 | 1.58±0.31 |
| Aurasperon B | 6 | 5.3 | 56490-522800 | 194261.67±176416.6 | 1 | 2.1 | 119960.0-119960.0 | 119960.0±0 |
| Aurasperon C | 8 | 7 | 27112-1523000 | 439875.25±471771.85 | 3 | 6.3 | 21484-700000 | 276668±369190 |
| Aurasperon G | 6 | 5.3 | 119200-2063000 | 546183.3±749954 | 1 | 2.1 | 114800-114800 | 114800±0 |
| Fonsecin | 4 | 3.5 | 0.42-4.6 | 1.69±1.95 | 7 | 14.6 | 0.4-93.1 | 14.3±34.8 |
| Malformin A | 6 | 5.3 | 0.73-3.91 | 1.92±1.25 | 5 | 10.4 | 0.8-59.4 | 19.6±23.7 |
| Malformin C | 5 | 4.4 | 0.72-1.73 | 1.24±0.42 | 5 | 10.4 | 0.9-14.3 | 6.4±5.7 |
| Nigragillin | 34 | 29.8 | 18144-7690000 | 1319818.06±2226963.78 | 17 | 35.4 | 40680-133720000 | 8808858±32207963 |
| Phenopyrrozin | 8 | 7 | 2.03-137.6 | 23.06±46.84 | 10 | 20.8 | 1.55-19 | 10.1±7.18 |
| Pyranonigrin | 0 | 0 | <LOD | <LOD | 1 | 2.1 | 3068.27 | 3068.3±0 |
| Butyrolactone III | 0 | 0 | <LOD | <LOD | 1 | 2.1 | 3.89 | 3.89±0 |
| Cyclopiazonic acid | 5 | 4.4 | 21.03-111.53 | 53.53±36.3 | 10 | 20.8 | 10.74-480 | 141.69±161.4 |
| Deoxyfunicone | 0 | 0 | <LOD | <LOD | 1 | 2.1 | 0.68-0.68 | 0.68±0 |
| Dichlordiaportin | 29 | 25.4 | 3.56-174.17 | ±40.12 | 12 | 25 | 4.39-95.3 | 33.99±32.6 |
| Kotanin A | 0 | 0 | <LOD | <LOD | 1 | 2.08 | 12.84-12.84 | 12.84±0 |
| Methylfunicone | 0 | 0 | <LOD | <LOD | 2 | 4.17 | 0.11-0.18 | 0.14±0.05 |
| Mevinolin | 0 | 0 | <LOD | <LOD | 5 | 10.42 | 1.68-22.33 | 9.51±8.44 |
| Sydonic acid | 1 | 0.9 | 1.05-1.05 | 1.05±0 | 3 | 6.25 | 1.05-2.87 | 2.17±0.98 |
| Sydonol | 2 | 1.8 | 0.81-4 | 2.40±2.25 | 5 | 10.42 | 2.63-20.64 | 6.81±7.75 |
| Terrein | 0 | 0 | <LOD | <LOD | 4 | 8.33 | 17.86-1083 | 295.58±525.35 |
| 1-Deoxypebrolide | 0 | 0 | <LOD | <LOD | 1 | 2.08 | 1.29-1.29 | 1.29±0 |
| Agroclavine | 15 | 13.2 | 0.11-527.1 | 67.56±176.6 | 6 | 12.5 | 0.12-1.73 | 0.70±0.66 |
| Andrastin A | 9 | 7.9 | 0.83-25.79 | 6.03±7.69 | 6 | 12.5 | 8.64-147.5 | 56.83±59.0 |
| Andrastin B | 2 | 1.8 | 17.96-20.4 | 19.17±1.71 | 4 | 8.33 | 13.42-195.0 | 79.51±80.81 |
| Andrastin C | 1 | 0.9 | 67.01-67.0 | 67.01±0 | 3 | 6.25 | 61.33-424.6 | 213.00±189 |
| Atpenin A5 | 2 | 1.8 | 1.32-1.56 | 1.44±0.17 | 0 | 0 | <LOD | <LOD |
| Barceloneic acid | 23 | 20.2 | 2.13-35.15 | 12.05±10.11 | 7 | 14.58 | 3.58-151.46 | 37.42±56.72 |
| Berkedrimane B | 0 | 0 | <LOD | <LOD | 3 | 6.25 | 3.55-59.12 | 26.51±29.0 |
| Bilaid A | 9 | 7.9 | 0.13-8.3 | 3.13±3.41 | 1 | 2.08 | 1.74 | 1.74±0 |
| Chaetominine | 2 | 1.8 | 2.25-14.44 | 8.34±8.62 | 4 | 8.33 | 8.55-38.03 | 23.22±15.98 |
| Chanoclavin | 15 | 13.2 | 0.04-36.35 | 5.07±11.47 | 9 | 18.75 | 0.04-15.62 | 2.69±5.23 |
| Chloromonilic acid B | 0 | 0 | <LOD | <LOD | 1 | 2.08 | 0.93-0.93 | 0.93±0 |
| Citreohybridinol | 18 | 15.8 | 1.10-409.53 | 52.28±103.32 | 10 | 20.83 | 0.61-1109.72 | 196.74±359.33 |
| Citreoviridin | 1 | 0.9 | 3.74-3.74 | 3.74±0 | 1 | 2.08 | 2.42 | 2.42±0 |
| Curvularin | 58 | 50.9 | 0.44-153.64 | 17.89±31 | 14 | 29.17 | 1.18-68.13 | 23.00±26.25 |
| Cyclopenin | 0 | 0 | <LOD | <LOD | 0 | 0 | <LOD | <LOD |
| Dechlorogriseofulvin | 1 | 0.9 | 0.36-0.36 | 0.36±0 | 2 | 4.17 | 5.49-70.44 | 37.96±45.93 |
| Demethylsulochrin | 0 | 0 | <LOD | <LOD | 5 | 10.42 | 3.74-30 | 12.61±10.2 |
| Deoxygerfelin | 2 | 1.8 | 0.21-0.59 | 0.40±0.3 | 9 | 18.75 | 0.12-13.70 | 1.88±4.45 |
| Dihydrocitrinone | 2 | 1.8 | 1.85-9.85 | 5.85±5.66 | 1 | 2.08 | 13 | 12.99±0 |
| Elymoclavine | 6 | 5.3 | 0.83-244.11 | 76.57±116.83 | 0 | 0 | <LOD | <LOD |
| Epoxyagroclavin | 0 | 0 | <LOD | <LOD | 1 | 2.08 | 9.72 | 9.72±0 |
| Flavoglaucin | 27 | 23.7 | 2.88-83.44 | 17.56±22.80 | 5 | 10.4 | 3.03-57.35 | 18.35±22.4 |
| Griseofulvin | 0 | 0 | <LOD | <LOD | 2 | 4.2 | 15.43-150.19 | 82.81±95.3 |
| Griseophenone A | 0 | 0 | <LOD | <LOD | 1 | 2.1 | 0.95-0.95 | 0.95±0 |
| Griseophenone B | 0 | 0 | <LOD | <LOD | 2 | 4.2 | 24.84-254.50 | 139.67±162.4 |
| Griseophenone C | 0 | 0 | <LOD | <LOD | 2 | 4.2 | 0.79-8.05 | 4.42±5.14 |
| Hydroxyandrastin A | 1 | 0.9 | 7.54 | 7.54±0 | 0 | 0 | <LOD | <LOD |
| Hydroxyandrastin C | 1 | 0.9 | 4.73 | 4.73±0 | 0 | 0 | <LOD | <LOD |
| Isosulochrin | 1 | 0.9 | 0.19 | 0.19±0 | 1 | 2.1 | 0.56 | 0.56±0 |
| Moniliphenone | 19 | 16.7 | 0.12-1.74 | 0.54±0.47 | 7 | 14.6 | 0.82-41.36 | 12.65±15.94 |
| Mycophenolic acid | 2 | 1.8 | 1.16-1.41 | 1.3±0.18 | 1 | 2.1 | 320.7 | 320.68±0 |
| O-Desmethyl-Mycophenolic acid | 0 | 0 | <LOD | <LOD | 1 | 2.1 | 3.67 | 3.67±0 |
| Oxaline | 3 | 2.6 | 0.17-0.32 | 0.26±0.08 | 3 | 6.3 | 0.16-0.66 | 0.33±0.29 |
| Oxidized Elymoclavine | 2 | 1.8 | 3.77-4.12 | 3.94±0.25 | 0 | 0 | <LOD | <LOD |
| Penicillic acid | 0 | 0 | <LOD | <LOD | 1 | 2.1 | 18.26 | 18.26±0 |
| Pinselin | 6 | 5.3 | 0.95-11.19 | 4.45±3.98 | 6 | 12.5 | 1.16-70.3 | 16.21±26.75 |
| Quinadoline A | 1 | 0.9 | 1.46 | 1.46±0 | 0 | 0 | <LOD | <LOD |
| Quinolactacin A | 43 | 37.7 | 0.02-2.89 | 0.22±0.49 | 12 | 25 | 0.02-59.6 | 5.38±17.1 |
| Rugulovasine A | 4 | 3.5 | 2.49-36.4 | 14.14±15.98 | 1 | 2.08 | 2.3 | 2.28±0 |
| Scalusamid A | 2 | 1.8 | 0.28-0.93 | 0.60±0.46 | 1 | 2.08 | 74 | 73.88±0 |
| Sclerotinin A | 3 | 2.6 | 0.48-4.74 | 2.48±2.14 | 5 | 10.42 | 0.61-2.48 | 1.25±0.77 |
| Viridicatum toxin | 0 | 0 | <LOD | <LOD | 3 | 6.3 | 196.68-1362 | 625.32±640.8 |
| Alternariol | 16 | 14 | 0.29-4.93 | 1.34±1.37 | 4 | 8.33 | 0.40-0.85 | 0.58±0.20 |
| Altersetin | 6 | 5.3 | 2.36-15.72 | 7.97±5.25 | 2 | 4.2 | 2.23-2.54 | 2.39±0.22 |
| Macrosporin | 61 | 53.5 | 0.37-38.8 | 4.55±8.38 | 7 | 14.6 | 0.40-1.44 | 0.94±0.37 |
| Pyrenophorol | 3 | 2.6 | 2.33-7.26 | 4.61±2.49 | 3 | 6.3 | 1.73-2.60 | 2.23±0.45 |
| Radicinin | 16 | 14 | 2.07-12.4 | 4.88±2.8 | 11 | 22.9 | 10.22-319.30 | 89.32±94.17 |
| Tentoxin | 39 | 34.2 | 0.95-9.26 | 2.75±2.08 | 9 | 18.8 | 1.00-2.21 | 1.38±0.43 |
| Ascochlorin | 6 | 5.3 | 0.39-14.79 | 6.25±5.65 | 5 | 10.4 | 0.32-10.23 | 2.87±4.20 |
| Cercosporin | 2 | 1.8 | 2.66-2.66 | 2.66±0.00 | 0 | 0 | <LOD | <LOD |
| Chlorocitreorosein | 1 | 0.9 | 1.48-1.48 | 1.48±0 | 1 | 2.1 | 2.37 | 2.37±0 |
| Chloronectrin | 2 | 1.8 | 0.43-0.59 | 0.51±0.12 | 2 | 4.2 | 0.23-0.53 | 0.38±0.21 |
| Cylindrocarpon A4 | 5 | 4.4 | 0.05- | 0.17± | 3 | 6.3 | 0.02-0.30 | 0.13±0.15 |
| Cylindrol B | 2 | 1.8 | 0.06-0.13 | 0.09±0.05 | 1 | 2.1 | 0.05 | 0.05±0 |
| Harzianopyridine | 1 | 0.9 | 1.69-1.7 | 1.69±0 | 0 | 0 | <LOD | <LOD |
| Heptelidic acid | 1 | 0.9 | 3.18-3.18 | 3.18±0 | 11 | 22.9 | 2.41-42.28 | 16.96±13.80 |
| Ilicicolin A | 0 | 0 | <LOD | <LOD | 1 | 2.1 | 0.95 | 0.95±0 |
| Ilicicolin B | 10 | 8.8 | 0.42-4.17 | 1.89±1.34 | 8 | 16.7 | 0.64-6.23 | 2.20±1.79 |
| Ilicicolin C | 4 | 3.5 | 2.07-5.93 | 3.97±1.89 | 1 | 2.1 | 6.78 | 6.78±0 |
| Ilicicolin E | 4 | 3.5 | 0.39-1.47 | 0.83±0.47 | 1 | 2.1 | 0.81 | 0.81±0 |
| Ilicicolin F | 4 | 3.5 | 0.16-0.56 | 0.38±0.19 | 2 | 4.2 | 0.26-0.40 | 0.33±0.10 |
| Ilicicolin H | 11 | 9.6 | 2.16-35.88 | 7.36±9.65 | 6 | 12.5 | 1.94-26.2 | 11.07±11.02 |
| LL-Z 1272e | 2 | 1.8 | 0.10-0.19 | 0.15±0.07 | 1 | 2.1 | 0.11 | 0.11±0 |
| Monocerin | 92 | 80.7 | 0.30-271.83 | 24.40±54.05 | 31 | 64.6 | 0.26-190.2 | 33.72±59.22 |
| Radicicol | 0 | 0 | <LOD | <LOD | 2 | 4.2 | 0.87-1.31 | 1.09±0.31 |
| Roridin A | 1 | 0.9 | 8.45-8.45 | 8.45±0 | 1 | 2.1 | 7.24 | 7.24±0 |
| Roridin L2 | 1 | 0.9 | 242.6 | 242.56±0 | 1 | 2.1 | 172.4 | 172.40±0 |
| Monactin | 4 | 3.5 | 0.78-1.1 | 0.96±0.13 | 6 | 12.5 | 0.47-4.91 | 2.97±1.83 |
| Nonactin | 7 | 6.1 | 0.03-0.23 | 0.15±0.07 | 7 | 14.6 | 0.08-0.93 | 0.41±0.34 |
| Ononin | 0 | 0 | <LOD | <LOD | 2 | 4.2 | 11.75-22.02 | 16.88±7.3 |
| Senkirkin | 15 | 13.2 | 0.57-2 | 1.00±0.38 | 4 | 8.3 | 1.01-1.8 | 1.35±0.4 |
| Asperglaucide | 41 | 36 | 0.20-34 | 2.59±6.63 | 13 | 27.1 | 0.25-23.24 | 2.85±6.3 |
| Chrysophanol | 2 | 1.8 | 12.34-26.34 | 19.34±9.90 | 3 | 6.3 | 3.51-8.2 | 6.62±2.7 |
| Citreorosein | 12 | 10.5 | 1.51-26.7 | 6.18±8.44 | 8 | 16.7 | 2.67-13.8 | 5.62±3.73 |
| cyclo(L-Pro-L-Tyr) | 113 | 99.1 | 4.10-470.4 | 61.28±63.7 | 47 | 98 | 3.20-1030 | 55.67±148.2 |
| cyclo(L-Pro-L-Val) | 110 | 96.5 | 3.30-271 | 50.37±44.6 | 45 | 93.8 | 2.40-637.6 | 45.47±96.4 |
| Endocrocin | 2 | 1.8 | 82.22-84.0 | 83.12±1.3 | 3 | 6.3 | 47.14-176 | 92.03±72.7 |
| Fellutanine A | 32 | 28.1 | 4.21-31.8 | 10.89±7.15 | 6 | 12.5 | 4.14-12.26 | 6.84±3.12 |
| Lecanoric acid | 9 | 7.9 | 0.40-1.6 | 0.84±0.5 | 7 | 14.6 | 1.95-10.31 | 4.50±3.1 |
| Methylorsellinic acid | 1 | 0.9 | 36.97-4 | 36.97±0 | 2 | 4.2 | 27.11-108.92 | 68.02±6 |
| Norlichexanthone | 1 | 0.9 | 0.26-0.3 | 0.26±0 | 2 | 4.2 | 1.22-10.86 | 6.04±6.82 |
| Oxyskyrin | 2 | 1.8 | 1.25-1.44 | 1.34±0.13 | 2 | 4.2 | 1.28-1.71 | 1.50±0.30 |
| Skyrin | 15 | 13.2 | 0.45-69.7 | 10.16±17.7 | 6 | 12.5 | 0.54-90.7 | 23.06±34.7 |

Metabolites concentration in µg/kg; n-Number of samples analyzed; np-number of positive samples; %p-Percent positive samples; Mean-positive samples only; SD-standard deviation; <LOD-Less than limit of detection
