# Supplementary material for: Mycotoxin exposure through the consumption of processed cereal food for children (< 5 years old) from rural households of Oshana, a region of Namibia
Source: Mycotoxin Res. 2025 Jan 14;41(1):249–65. doi: 10.1007/s12550-024-00580-z (PMC11759469; doi:10.1007/s12550-024-00580-z)
Supplement: Supplementary file 2 — Supplementary file2 (DOCX 17 KB) [file 12550_2024_580_MOESM2_ESM.docx]

**Table S2: Occurrence of major mycotoxins and their derivatives in all the processed cereal food samples (n=162) from Oshana region.**

| Metabolite type | Np | %p | Range | Mean±SD |
| --- | --- | --- | --- | --- |
| Aflatoxin B_1_ | 58 | 35.8 | 0.16-40.10 | 3.07±6.2 |
| Aflatoxin B_2_ | 10 | 6.2 | 0.24-1.02 | 0.49±0.3 |
| Aflatoxin G_1_ | 4 | 2.5 | 0.19-1.53 | 0.61±0.6 |
| Total aflatoxin | 58 | 35.8 | 0.16-41.12 | 1.17±4.2 |
| Aflatoxin M_1_ | 4 | 2.5 | 0.59-1.75 | 1.05±0.6 |
| Fumonisin A_1_ | 14 | 8.6 | 0.73-46.75 | 9.35±13 |
| Fumonisin A_2_ | 3 | 1.9 | 1.57-35.6 | 16.88±17.3 |
| Fumonisin B_1_ | 39 | 24.1 | 8.43-1022 | 143.65±227.4 |
| Fumonisin B_2_ | 25 | 15.4 | 8.22-524 | 80.81±118 |
| Fumonisin B_3_ | 14 | 8.6 | 12.88-163.1 | 54.48±43.6 |
| Fumonisin B_4_ | 13 | 8.0 | 10.15-157.6 | 41.74±43.1 |
| Total fumonisin | 40 | 24.7 | 8.43-1949 | 56.23±220.8 |
| Zearalenone | 44 | 27.2 | 0.25-40.16 | 4.62±8.1 |
| Deoxynivalenol | 17 | 10.5 | 14.03-202.7 | 82.65±60.5 |
| Nivalenol | 5 | 3.1 | 8.86-26.8 | 19.95±6.7 |
| Monoacetoxyscirpenol | 41 | 25.3 | 1.41-198.4 | 24.96±39 |
| Diacetoxyscirpenol | 13 | 8.0 | 1.5-50.6 | 8.65±13.5 |
| Neosolaniol | 6 | 3.7 | 1.9-11.56 | 5.28±4.0 |
| Deacetylneosolaniol | 6 | 3.7 | 16.5-215.2 | 86.72±69 |
| T2-Tetraol | 6 | 3.7 | 10.5-74.7 | 28.97±24.1 |
| 8-Acetylneosolanol | 18 | 11.1 | 0.29-60.8 | 11.45±17.3 |
| Citrinin | 20 | 12.3 | 23.85-9703 | 712.69±2137 |

Metabolites concentration in µg/kg; n-Number of samples analyzed; np-number of positive samples; %p-Percent positive samples; Mean-positive samples only; SD-standard deviation
